# Mismatch repair disturbs meiotic crossover control in *S. cerevisiae*

**DOI:** 10.1093/nar/gkaf1136

**Published:** 2025-11-13

**Authors:** Jon A Harper, Tim J Cooper, Margaret R Crawford, Laura J Hunt, Rachal M Allison, Marie-Claude Marsolier-Kergoat, Bertrand Llorente, Matthew J Neale

**Affiliations:** Genome Damage and Stability Centre, School of Life Sciences, University of Sussex, BN1 9RQ, United Kingdom; Genome Damage and Stability Centre, School of Life Sciences, University of Sussex, BN1 9RQ, United Kingdom; Genome Damage and Stability Centre, School of Life Sciences, University of Sussex, BN1 9RQ, United Kingdom; Genomics Core Facility, Francis Crick Institute, London, NW1 1AT, United Kingdom; Genome Damage and Stability Centre, School of Life Sciences, University of Sussex, BN1 9RQ, United Kingdom; School of Applied Sciences, University of Brighton, Brighton, BN2 4GJ, United Kingdom; Genome Damage and Stability Centre, School of Life Sciences, University of Sussex, BN1 9RQ, United Kingdom; Institute for Integrative Biology of the Cell (I2BC), CEA, CNRS, Univ. Paris-Sud, Université Paris-Saclay, 91190 Gif-sur-Yvette, France; UMR7206 Eco-Anthropology and Ethno-Biology, CNRS-MNHN-University Paris Diderot, Musée de l’Homme, 75016 Paris, France; Cancer Research Centre of Marseille, CNRS, INSERM U1068, Institut Paoli-Calmettes, Aix-Marseille Université UM105, 13273 Marseille, France; Genome Damage and Stability Centre, School of Life Sciences, University of Sussex, BN1 9RQ, United Kingdom

## Abstract

Crossover formation during meiosis generates genetic diversity. In many species most crossovers display interference, meaning they are spaced more evenly than expected by chance, and are called class I crossovers. Class II crossovers, a minority pathway, are believed to lack substantial interference. Here, using whole-genome recombination maps, we examine the impact of mismatch repair (MMR) on the formation and distribution of crossovers in *Saccharomyces cerevisiae*. Loss of the MMR protein Msh2 increases the uniformity of crossover distributions—an effect that is independent of changes in crossover frequency. Simulations indicate that this effect is driven by increases in the class I crossover proportion without any change in interference strength. Consistent with this view, distributions of Zip3 foci, specific markers of class I crossovers, are unchanged by *MSH2* deletion. Notably, in wild-type cells, fewer crossovers arise in regions of higher polymorphism density—a skew that depends on both Msh2 and Zip3. Taken together, our results indicate a dual influence of Msh2 on recombination: suppression of class I crossovers in regions of higher polymorphism density, whilst unexpectedly promoting class II crossover formation. Our findings highlight how MMR shapes the landscape of genetic exchange, and links recombination to sequence divergence and its role in speciation.

## Graphical Abstract

**Graphical Abstract:**
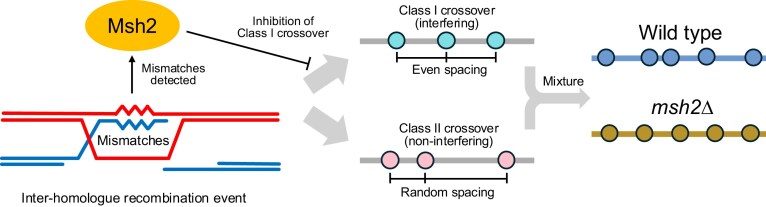

## Background

Meiosis is a key process in reproduction, generating genetically diverse gametes through the process of recombination. The generation of DNA double-strand breaks (DSBs) by Spo11 is critical for the initiation of homologous recombination [1]. Repair of such DSBs can result in either crossovers (COs)—in which homologous chromosomes exchange DNA—or noncrossovers, where DNA is repaired without any exchange between homologous chromosomes [2, 3]. In many organisms, COs are essential for correct segregation of meiotic chromosomes during anaphase I, chromosomal abnormalities such as aneuploidy becoming much more likely when any chromosomes lack a CO [4–8]. There is therefore significant selective pressure to ensure that every chromosome within the genome possesses at least one CO.

Within many organisms, including *Saccharomyces cerevisiae* (the subject of this study), *Mus musculus, Homo sapiens*, and *Arabidopsis thaliana*, two subclasses of CO co-exist. ZMM (Zip1–Zip2–Zip3–Zip4–Spo16, Msh4–Msh5, Mer3)- and Mlh1–Mlh3-dependent class I COs account for the majority of COs formed (∼70%–85% within *S. cerevisiae*) [9, 10]. Class I COs are distributed more evenly along each chromosome than expected by chance via a process referred to as CO interference in which the formation of COs in proximity to one another appears suppressed (for review [3]). Class I CO formation requires the nuclease activity of Mlh1–Mlh3, a heterodimer otherwise involved in mismatch repair (MMR) [11–15]. The less abundant class II COs are generally considered noninterfering, displaying a more random distribution, and their formation depends upon the Mus81–Mms4, Yen1, or Slx1–Slx4 structure-specific nucleases [10, 16–18].

Current models of class I CO formation suggest at least a two-step process involving, initially, the designation of a subset of precursor DSB intermediates by pro-class I CO factors [19]. Although CO interference is proposed to be active at this stage, thereby shaping the distribution of COs along chromosomes, the final distribution of class I events is secondarily impacted by a later process: class I CO maturation [20]. Rates of maturation <100% can lead to achiasmatic chromosomes, or chromosomes with a residual CO positioned close to one or other telomere, where they are considered an “at-risk” location for successful segregation [21]. Such ideas have been developed to help explain the relatively high rate of meiotic chromosome missegregation in human females relative to males [21].

For homologous recombination to take place, repair templates require near-perfect sequence identity with the invading strand [22]. When present, mismatches are detected through the action of MMR proteins such as Msh2, therefore mismatch recognition becomes an integral part of recombination. Msh2 is essential for MMR (orthologous to the bacterial MutS) [23], binding mismatches and promoting their repair via endonucleolytic action of the Mlh1–Pms1 and Mlh1–Mlh3 complexes [24, 25]. Msh2 acts as a heterodimer, with either of two binding partners: Msh6 for Single Nucleotide Polymorphisms (SNPs) [26] and Msh3 for insertion-deletions (indels) [27]. Though important, Msh2-dependent processing may lead to the formation of complex lesions during MMR [28, 29]. Msh2 also participates in flap cleavage independently of mismatch recognition itself [30]. Msh2-dependent recognition of mismatches that arise during recombination may also lead to rejection of the nascent recombination event—independently of any direct repair of mismatched sequences [30].

All of the functions of MMR discussed above have the potential to impact local and global recombination patterns. Indeed, MMR-proficient *S. cerevisiae* hybrids display inefficient homologous recombination, leading to reduced rates of meiotic CO formation, reduced spore viability, and increased chromosomal nondisjunction during meiosis I [31, 32]—phenotypes linked to incipient speciation, and which are largely reversed within MMR-deficient strains [31–36]. However, whilst Msh2-dependent binding to mismatches is likely to be rapid [26], the precise stage of the HR pathway(s) that mismatch recognition arises, and whether this is the same for all intermediates, and in all organisms, is unclear. Indeed, recent observations in mouse meiosis suggest only limited impact of MMR activity on recombination suppression [37], and perhaps more intriguingly, observations in *A. thaliana* indicate an unexpected association—rather than inhibition—of recombination within polymorphic regions [38]. Given the fundamental intimate relationships between MMR, sequence divergence, and meiotic recombination, such observations highlight the need to thoroughly explore, and to understand better, the impacts that meiotic MMR activity may have across biology.

Here, we investigate the effects of Msh2 on CO frequency and distribution in meiotic *S. cerevisiae* cells. We provide evidence that Msh2 activity induces preferential failure of class I COs in regions of higher polymorphism density, whilst stabilising class II CO, resulting in more variable CO distributions along and between chromosomes. Msh2’s anticrossover activity is dependent on Zip3, whereas Msh2 may play an unexpected role in promoting CO formation when polymorphisms are present.

## Materials and methods

### Yeast strains

All *S. cerevisiae* strains used in this study are derivatives of SK1 and S288c. Hybrid strains, utilized in genome-wide mapping, were derived from a cross of haploid SK1 and S288c. Strain genotypes are detailed in (Supplementary Table 1). Knockouts were performed and tested by standard transformation and polymerase chain reaction (PCR) techniques. *msh2*Δ*::kanMX6* and *zip3*Δ*::HphMX* were generated by PCR mediated gene replacement using a pFA6a-kanMX6 or pFA6-hphMX plasmid. The *P_GAL1_NDT80::TRP1* allele has the natural *NDT80* promoter replaced by the *GAL1-10* promoter, and strains include a *GAL4::ER* chimeric transactivator for β-estradiol-induced expression. S288c x SK1 hybrids create viable spores (81.98% wild type, 72.99% *msh2*Δ, 70.35% *ndt80AR*, 73.21% *msh2*∆ *ndt80AR* spore viability; Supplementary Table 2) [39], though it should be noted that observational bias may arise from assaying only the four-spore viable population.

### Meiotic timecourse (*ndt80AR* strains)

Diploid strains were incubated at 30°C on YPD plates for 48 h. For SK1 diploids, a single colony is inoculated into 4 ml YPD (1% yeast extract, 2% peptone, 2% glucose) and incubated at 30°C at 250 rpm for 24 h. For hybrid crosses, haploid parental isolates were mated in 1 ml YPD for 8 h. An additional 3 ml of YPD was subsequently added and the cells were grown for 16 h. Cells were inoculated to a density of (OD600) 0.2 into 30 ml YPA (1% yeast extract, 2% peptone, 1% K-acetate) and incubated at 250 rpm at 30°C for 14 h. Cells were collected by centrifugation, washed in H_2_O, and resuspended in 30ml pre-warmed sporulation media (2% potassium acetate, 5 μg/ml adenine, 5 μg/ml arginine, 5 μg/ml histidine, 15 μg/ml leucine, 5 μg/ml tryptophan, 5 μg/ml uracil). The culture was then incubated at 30°C at 250 rpm for the duration of the time course. After 8 h, 2 ml of the synchronized cultures were split and exposed to β-estradiol to a final concentration of 2 mM, which induces the transcription of *NDT80* and thus sporulation. Cultures were then incubated to a total of 48 h at 30°C prior to dissection.

### Tetrad dissection

In order to produce hybrid spores for sequencing, SK1 x S288c haploid parents were mated for 8–14 h on YPD plates, with the exception of *ndt80AR* strains, which are mated and grown in liquid YPD for 24 h (see above). Haploids were mated freshly on each occasion and not propagated as diploids, in order to reduce the potential for mutations accumulating. Sporulation was induced, and tetrads were dissected after 72 h in 2% potassium acetate. For octads, spores were additionally grown for 4–8 h on YPD plates until a single mitotic division was completed, after which the mother-daughter pair were separated.

### NGS library preparation

Spore colonies were grown for 16 h within liquid YPD for genomic DNA extraction. Only tetrads and octads producing four or eight viable spores/colonies, respectively, were considered for genotyping by NGS. Genomic DNA was purified from overnight saturated YPD cultures using standard phenol-chloroform extraction techniques. Samples of genomic DNA were diluted to 0.2–0.3 ng/μl. DNA concentration was measured using the Qubit High Sensitivity double-stranded DNA Assay. Genomic DNA was fragmented, indexed and amplified via the Nextera XT DNA library Prep Kit according to the best practices recommended by Illumina. In order to check fragment length distribution and concentration of purified libraries, 1 μl of undiluted library was run on an Agilent Technology 2100 Bioanalyzer using a High Sensitivity DNA chip. To pool samples for sequencing, 5 μl of each sample was combined into a 1.5 ml tube and mixed. Twenty-four microlitres of the mix was transferred to a tube containing 570 μl hybridisation buffer. The mix was boiled at 96°C for 2 min and placed in ice water for 5 min. Six microlitres of denatured PhiX control (prepared according to Illumina protocol, final concentration 1%) was added to the library, mixed well and then loaded into a MiSeq reagent cartridge. Sequencing was performed in-house using Illumina MiSeq instrument with paired-end 2 × 300 bp V3 reagents (Illumina).

### SNP and indel detection

Individual spores were sequenced to an average read-depth of ∼45×. Initially, paired-end read FASTQ files were aligned, via Bowtie2, to the SacCer3 reference genome (v. R64-2-1) using the parameters: -X 1000 —local —mp 5,1 -D 20 -R 3 -N 1 -L 20 -i S,1,0.50. In order to create a custom SK1 genome to facilitate more accurate genotype-calling, SNP, and indel polymorphisms were detected using the GATK (GenomeAnalysisToolkit) function HaplotypeCaller. An in-house script (VariantCaller.pl) subsequently parsed the resulting VCF files from 72 spores to calculate: (i) the call frequency (% of spores any given allele is present within), (ii) the cumulative allelic read depth (% of reads that contain a specific allele at a specific loci), and (iii) the cumulative total read depth. To identify legitimate SNPs and indels, variants were filtered for a call-frequency between 44% and 55%, a total read depth of >250, and an allelic read depth of 95%. Variants within repeat regions, long terminal repeats, retrotransposons and telomeres were also discarded—yielding a final, robust list of 64 591 SNPs and 3972 indels amounting to ∼0.57% divergence. A custom SK1 genome (SK1_Mod) was then generated by modifying SacCer3 (v. R64-2-1) to include all these SNPs and indels.

### Genotype-calling

Spore data from individual samples was aligned to both the custom SK1_Mod genome and the SacCer3 reference (see below). Alignment produces a SAM file, which was converted into a sorted BAM file using the Samtools function, view, for downstream processing. The PySamStats (v. 1.0.1, Miles and Mattioni) module, variation, was used to process the sorted BAM file for each sequenced spore, producing a list of the number of reads containing A/C/T/G, insertion or deletion for each genomic position specified in the S288c and SK1 references. Variant reads were isolated and genotyped using in-house, custom scripts as follows. Genotypes are assigned according to the rules: (i) A minimum read-depth of 5; (ii) A SNP was called as having the variant genotype if ≥70% of the reads at that position match the called variant, or as reference if ≥90% of the reads match the reference; (iii) If the variant and reference reads are above 90% of all reads and within 70% of each other, the position was called as heteroduplex; (iv) indels are called as having the variant genotype if ≥30% of the reads at that position match the variant. Such a low threshold is utilized because alignment of indel sequences is biased towards the reference, which means that they are unlikely to be erroneously called as matching the variant genotype. For an indel to be called as the reference genotype, ≥95% of the reads must match the reference sequence and there must be fewer than two reads matching the variant call. Any variants that fall below these thresholds were discarded. Genotype calls are converted into a binary signal, either 1 for S288c or 0 for SK1.

### Event calling

Using the binarized input, chromosomes were split into segments with the same segregation pattern using published scripts [39, 40]. Segment types (i.e. 1:7, 2:6, 2:6, 3:5, 4:4, 4:4*, 5:3, 6:2, 6:2*, or 7:1 as described in [34, 40]) were also recorded. Recombination events were subsequently called as being a set of segments located between two 4:4 segments longer than 1.5 kb. A 4:4 segment corresponds to a Mendelian segregation profile, 5:3 and 3:5 segments to half-conversion (heteroduplex) tracts and 6:2 and 2:6 segments to full conversion tracts. Each recombination event can contain between 0 and 2 COs or noncrossovers (NCOs). Events are additionally classified by the number of chromatids involved (i.e. 1, 2, either sister or nonsister, 3, 4). To ensure compatibility with our data-analysis pipeline, published binarized input data (“segFiles’) from the S96 x YJM789 hybrid [41–44] were minimally processed to match column naming, with spores from tetrads each duplicated to create a fake “octad”. This conversion involved no changes in data, only minimal reformatting. Recombination events were then called in the same way as for SK1 x S288c octads generated for this study. Any small differences in CO and NCO counts and positions between the resulting data and that published are likely, therefore, to be due to subtle differences in the event calling criteria used (for example event merging thresholds).

### Calculating chromosomal density of COs and polymorphisms

To evaluate genome-wide CO and polymorphism densities, chromosomes were divided into arms based on the position of the centromere. The telomeric end of the chromosome arms was then assigned as position 0, and the centromeric end given position 1. Positions along the chromosome arms were then divided into bins of length 0.01, and COs (or polymorphisms) were counted across these bins. The frequencies per bin were then summed across all 32 chromosome arms. Densities were normalized by dividing by the mean bin density. CO densities were calculated independently for each strain. For polymorphisms, only markers used to detect recombination events were included; low confidence markers were excluded (as described in [39]).

### Calculation of inter-event distances

For the purposes of our analyses, the start of a recombination event was taken as the midpoint between the last event marker before the event and the first marker specific to the event. The end of an event was taken as the midpoint between the last marker specific to the event and the first marker past the event. Positions of events were then calculated to be the midpoint between the computed start and end positions of the event. Inter-event distances (IEDs) were calculated as the distance (in bp) between successive event midpoints.

### Transformation and gamma modelling

Prior to model fitting, inter-crossover distances (ICDs) were transformed by dividing by the mean ICD for each cell, such that the mean ICD post-transformation was 1. Gamma distributions were fitted to ICD populations using maximum-likelihood estimation, conducted in R (version 4.3) using the mixfit function from the mixR package [45]. Gamma distributions are described with two parameters: shape/alpha (α) and scale/beta (β). Both α and β can be calculated from the mean and standard deviation of the fitted distribution [46]. Transforming data by dividing by the mean value does not change the α of the fitted gamma distribution, only β. Furthermore, when mean = 1, α = 1/β. Transformed gamma distributions can therefore be described using either α or β. Gamma mixture models include the additional parameter of proportion, which describes an estimate of how many values in the overall distribution belong to each of the fitted gamma distributions.

### Generating data using gamma distributions

Samples from gamma models were taken using the rgamma function in the GammaDist package for R (version 4.3).

### Calculation of CoC

Coefficient of Coincidence (CoC) is a statistical test used to assess crossover interference [20]. Chromosomes were first divided into intervals of equal size. Frequencies of crossovers were then calculated for each interval. The expected frequency of coincidence for each pair of intervals was calculated by multiplying the crossover frequencies of the intervals together, thus assuming the occurrence of crossovers within the intervals is independent. The observed frequency of coincidence (the proportion of meioses in which crossovers occurred within both intervals) was divided by the expected frequency to calculate CoC. CoC values for every interval pair of equal distance were pooled and averaged to form a final CoC for every distance. To increase the power of this analysis, distances of equal value across all chromosomes were pooled.

### Simulating CO interference

Simulated ICD distributions were produced using a simulator built in R (version 4.3). The simulator generates virtual chromosomes of user-specified length and number, binning coordinates by user-specified bin widths (set as 1 kb wide for all data presented here). Each bin is assigned two relative probabilities—one for class I events, the other for class II events, both of which start as 1 in all bins. A bin is then drawn randomly based on these probabilities, which is designated as the bin in which a CO occurs.

The simulator acts on virtual chromosomes, of user specified number and length. Proportions of COs per chromosome can also be given, or weighted probabilities can be supplied to randomly allocate each CO to a chromosome. For the purposes of our analysis, we used the *S. cerevisiae* genome with proportions of COs allocated to each virtual chromosome based on those observed in our wild-type data.

If the generated event is a class I CO, a window of interference is applied, decreasing the probabilities of CO formation in adjacent bins in a manner where such repression decays with distance. Such inhibition thereby reduces or eliminates the chance that subsequent class I events will occur in such bins. No interference window is applied to class II COs. The probabilities in bins within a user-specified detection threshold (default 2 kb) are then reduced to 0, to emulate the inability of our sequencing method to distinguish COs occurring within 1.5 kb of each other.

To fit sequencing data, each simulation run continued until the total number of visible COs across all virtual chromosomes equalled that of a given genotype (e.g. 75 for wild type, 105 for *msh2*Δ). To minimize sampling issues, and ensure that the results presented are representative, simulations were repeated 1000 times for each combination of parameters (e.g. class II probability, CO failure rate).

### Simulation of CO maturation failure

We modelled CO maturation failure in two different ways. The first was by randomly failing class I COs as they were generated. The second was to generate a fixed number of failed COs before starting the simulation, effectively prepatterning chromosomes with patterns of interference generated by failed COs.

CO maturation failure was modelled such that the proportion of class II events represented the proportion of all CO events attempted rather than just the proportion of successful events (i.e. as a function of the sum total of class I, class II and failed COs, rather than only the successful class I and class II COs). This required running the simulator such that CO failure leads to re-designation of the next event, instead of simply re-attempting to form a class I CO (i.e the designation check is repeated, allowing the next CO to be class I or class II).

### Microscopy and cytological analysis

4.5 ml of meiotic culture was spun down on a bench centrifuge and resuspended to 500 μl with 1 M pH 7.0 D-Sorbitol. 12 μl of 1.0 M DTT and 7 μl of 10 mg/ml Zymolyase in 10% glucose solution was added and cells were spheroplasted by incubation at 37°C for 35–50 min with agitation. Spheroplasting success was determined by taking 2–3 μl of the aforementioned solution and adding an equivalent volume of 1.0% (w/v) sodium N-lauroylsarcosine while under microscopic observation; 3.5 ml of Stop Solution [0.1 M MES, 1 mM ethylenediaminetetraacetic acid (EDTA), 0.5 mM MgCl_2_, 1 M D-sorbitol, pH 6.4] was subsequently added and the cells were spun down and resuspended in 100 μl Spread Solution (0.1 M MES, 1 mM EDTA, 0.5 mM MgCl_2_, pH 6.4) and distributed between four slides, which had been soaked in 70% ethanol overnight and wiped clean.

To each slide, fixative [4.0% (w/v) formaldehyde, 3.8% (w/v) sucrose, pH 7.5] was added dropwise, followed by detergent (1% Lipsol, 0.1% Bibby Sterilin) to a ratio of 1:3:6 (suspension:fixative:detergent) before lightly mixing and incubating for 1 min at room temperature (RT). Further fixative was added dropwise to a final ratio of 1:9:6 and the mixture spread across the slide. Each spread was subsequently incubated at RT for 30 min in damp conditions, then allowed to air-dry at RT overnight. Once dry, slides were sequentially washed in 0.2% (v/v) PhotoFlo Wetting Agent (Kodak) and dH_2_O, and stored at 4°C.

Slides were washed once in 0.025% Triton X-100 for 10 m at RT and twice in phosphate buffered saline (PBS) for 5 min at RT. Slides were blocked in 5% skimmed milk with PBS for 3 h at 37°C. Excess liquid was removed and slides laid horizontally in damp conditions. Forty microlitres of primary antibody {anti-Zip3 from rat at 1:200 and anti-Red1 [Genecust, affinity purified, raised against aa(426–827)] from rabbit at 1:200} in 1% skimmed milk with PBS was added under coverslips. Slides were incubated at 4°C overnight (15.5 h) and washed three times in PBS for 5 min at RT. Excess liquid was then removed and slides were returned to damp conditions. Forty microlitres of secondary antibody (antirat AlexaFluor555 at 1:200 and antirabbit AlexaFluor488 at 1:500) in 1% skimmed milk with PBS was then added under coverslips. Slides were incubated at RT for 2.5 h and then washed three times with PBS for 5 min at RT. Cover slips were affixed using Vectashield mounting medium with DAPI, sealed with clear varnish and imaged on an Olympus IX71 (z = 0.2 μM, exposure times: TRITC-mCherry = 0.2 s, eGFP = 1.0 s, 4′,6-diamidino-2-phenylindole (DAPI) = 0.1 s). Images were randomized, deconvoluted via Huygens (software) and foci were automatically counted using an in-house plugin for ImageJ (FindFoci) as previously described [47] with an appropriate mask to discard signals outside of nuclei.

To measure inter-foci distances, pixels denoting the centre of each Zip3 focus and Zip1 ends were manually selected along clearly separable bivalents as determined by Zip1-GFP signal (120–156 bivalents per strain, error margin of ∼1 pixel = 0.1 µm). These positions were selected using the ImageJ segmented line tool. Segment lengths were then calculated by macro, confirming against total length as measured by ImageJ standard tool.

For the purposes of deconvolving overlapping or adjacent foci, raw z-stack images were processed with Hyugens (Scientific Volume Imaging). The point spread function was calculated by the Hyugens software, using default parameters. Deconvolution was performed using a standard algorithm with 50 iterations.

### Assessing polymorphism density

Polymorphism density was calculated by defining a region of length 1 kb centered on the midpoint of each CO/NCO and counting the number of SNPs within the region. Spo11-DSB hotspot data was used for the expected curve [48], representing CO/NCO formation unbiased by polymorphism density (i.e. the pattern of DSB formation prior to repair).

### Statistical analyses

A Kolmogorov–Smirnov (KS) goodness-of-fit (GoF) test is a nonparametric test and was used to compare continuous probability distributions of CO positions in order to assess the null hypothesis that both samples derive from identical populations, based on their maximal difference (D). *P*-values of the KS test effectively describe the probability that, if the null hypothesis is true, the observed CDFs would be as far apart as observed. *P*-values may therefore constitute an indirect measure of distributional agreement, as employed throughout this research. KS tests were performed using the ks.test function from the R stats package. All statistical analyses were performed in R version 4.4.2.

## Results

### Loss of Msh2 activity increases meiotic CO frequency

**Figure 1.**
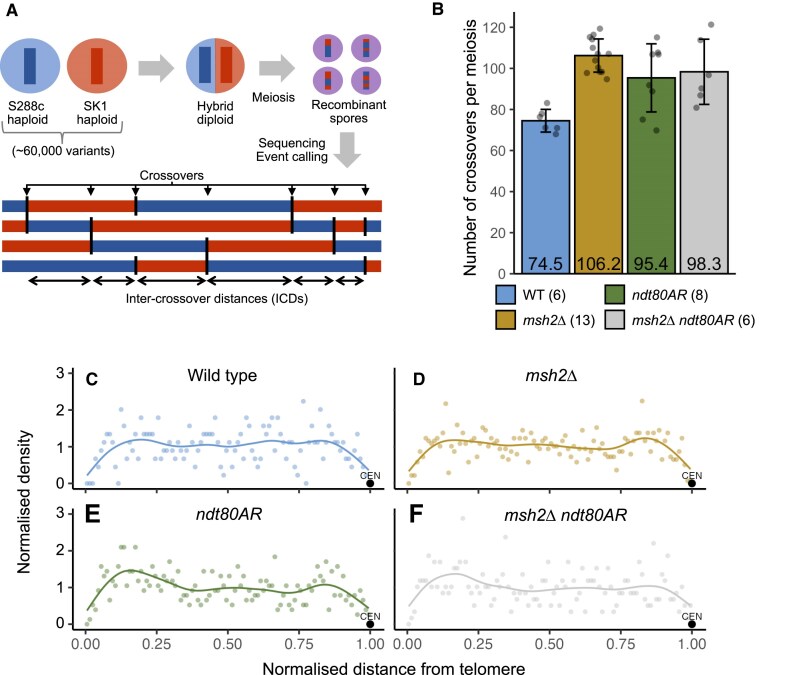
Crossover numbers are elevated in *msh2*∆ and *ndt80AR*. (**A**) Genome-wide mapping of recombination. Meiosis is induced within hybrid S288c × SK1 *S. cerevisiae* diploid cells and genomic material is prepared from individual, isolated spores for paired-end Illumina sequencing in order to genotype SNP/indel patterns and therefore determine the parental origin of any given locus (see the ‘Materials and methods’ section). The cartoon below depicts exchanges along a single chromosome. ICDs are calculated as the distance (in bp) between successive COs along a given chromosome. (**B**) Individual (grey circles) and average number (bars, and inset numbers) of COs per meiosis for each genotype. The number of individual meioses sequenced per genotype is indicated in brackets. Error bars represent standard deviation. (**C**–**F**) Crossover density across chromosome arms. Chromosomes were split either side of the centromere and CO positions expressed as proportion of distance from telomere, normalizing for the size of chromosome arms. Each point is the CO density in that bin averaged across all 32 arms. Densities were normalized by the mean density in each genotype.

To investigate the role that MMR has on recombination outcomes we characterized recently published datasets [39] in which meiotic CO and NCO events were mapped genome-wide in the budding yeast *S. cerevisiae* [39]. In total, six wild-type and thirteen MMR-defective *msh2*Δ meioses (Fig. 1A) were analysed from a cross of two widely utilized laboratory isolates: S288c and SK1 (∼0.57% divergence). Msh2 was chosen to abrogate both forms of MMR (both detection of SNPs and indels). Additionally, we reanalyzed datasets comprising fifty-one wild-type and four *msh2*Δ tetrads from a S96 x YJM789 cross of *S. cerevisiae* (∼0.6% divergence) [42]. On average, 74.5 ± 5.8 and 106.2 ± 4.9 COs per meiosis were detected within our SK1 × S288c wild-type and *msh2*Δ samples respectively, corresponding to a significant 1.4-fold increase in CO frequency (*P *<.001, Wilcoxon test, Fig. 1B). A significant *msh2*Δ-dependent increase was also observed within YJM × S96 hybrids (*P = *0.0012, two-sample KS test; Supplementary Fig. S1A)—collectively reaffirming the known antirecombinogenic activity of Msh2 [34, 49–51]. Notably, CO frequencies were considerably higher within wild type S96 × YJM789 than SK1 × S288c (92.0 versus 74.5 COs per wild type meiosis, *P *= 0.0003)—suggesting that cross-specific differences may exist (Fig. 1B and Supplementary Fig. S1A).

Deletion of *MSH2* also increased observed NCO events in both hybrid crosses (SK1 × S288c: wild type = 30.5; *msh2*∆ = 92.4; YJM × S96: wild type = 46.8; *msh2*∆ = 56.2; Supplementary Fig. S1B and C), supporting the established effect of Msh2 in suppressing both COs and NCOs in hybrid strains as reported previously [34]. However, unlike COs, the visibility of NCOs is directly affected both by the true number of converted and/or heteroduplex markers contained within a NCO event, and by the technical efficiency of calling what may potentially be only short regions of contiguous nonreciprocal marker change. NCO frequencies are also more likely to be affected by homeostatic effects than are COs [5, 34, 39]. For these reasons, quantitative comparisons of NCO frequency changes in the presence and absence of *MSH2* are not simple to interpret. Instead, we focused our attention on COs, where event detection is assumed to be similar ± Msh2, yet nonetheless displays a substantial increase in frequency upon *MSH2* deletion.

### Spatial distributions of COs are altered upon *MSH2* deletion

To investigate potential effects of Msh2 on CO patterning associated with the increased CO frequency, we first sought to determine if distributions of COs were altered along chromosomes. To this end, we plotted the average CO density as a function of distance after normalizing the scale of chromosome arms between the centromere and telomere (Fig. 1C). Across the meioses studied, COs were relatively uniformly distributed along chromosome arms but with regions of relative suppression close to telomeric and centromeric regions. In addition, subtelomeric and subcentromeric regions displayed a slight elevation in CO density (Fig. 1C). Such patterns were recaptured in the *msh2*∆ strain (Fig. 1D). Deletion of *MSH2* therefore does not appear to have an obvious effect on population-wide distributions of COs (Fig. 1C and D). Importantly, the genetic polymorphisms used to map the recombination events between the SK1 and S288c parent genomes are uniformly distributed, lack any such subtelomeric or subcentromeric enrichment, and are only uncommon in the most distal telomeric regions (Supplementary Fig. S1D). Similar CO density patterns to SK1 × S288C hybrids were observed in the S96 × YJM789 cross (Supplementary Fig. S1E and F).

**Figure 2.**
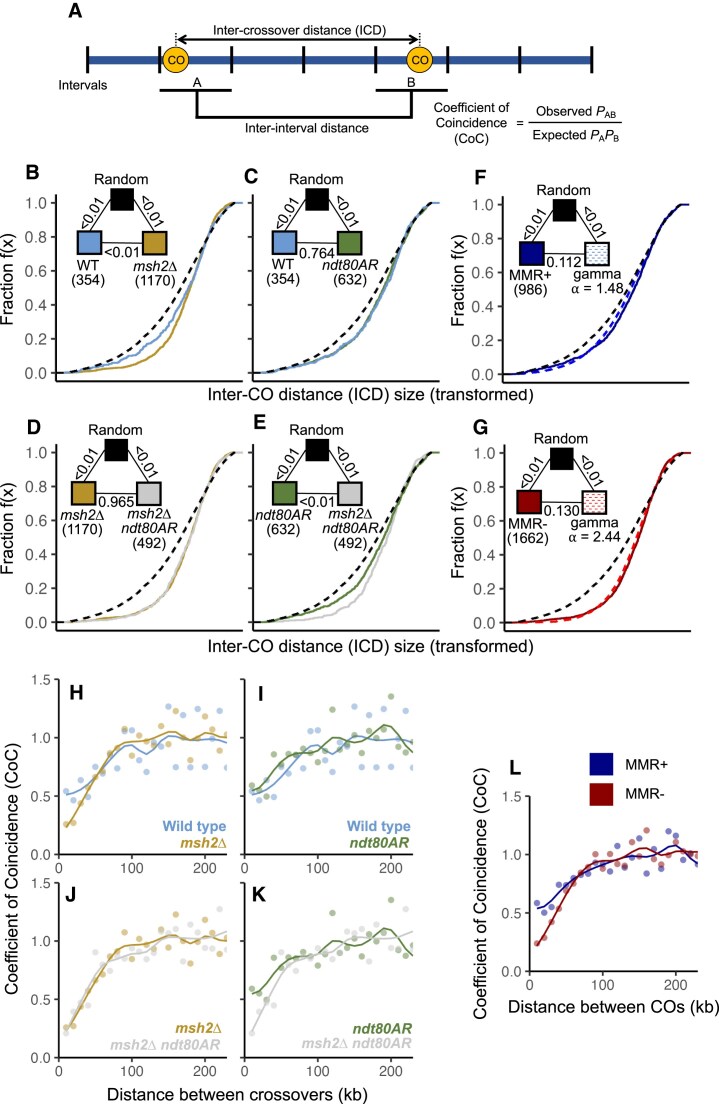
Crossover distributions are more uniform in the absence of Msh2. (**A**) Schematic to show calculation of ICD and coefficient of coincidence (CoC). (**B**–**G**) eCDFs showing the fraction of ICDs at or below a given size. ICDs are transformed by dividing by the mean to correct for skews generated by differing CO frequencies (see the ‘Materials and methods’ section). Black dashed lines are datasets generated via simulation to represent a random distribution (see the ‘Materials and methods’ section). Pairwise goodness-of-fit (GoF) tests were performed between genotypes as indicated (triangular legend). *P*-values: Two-sample KS test. Numbers in brackets indicate the total number of ICDs in each genotype. (**H**–**L**) CoC curves across the stated genotypes. Paired intervals of the same distance across different chromosomes were pooled and averaged to calculate CoC. In panel (L), MMR+ indicates pooled wild type and *ndt80AR* datasets. MMR− indicates pooled *msh2*∆ and *msh2*∆ *ndt80AR* datasets.

Though the population-averaged distribution of COs does not change upon *MSH2* deletion, such analysis cannot characterize differences that may exist in the positioning of COs relative to one another along individual chromosomes. To further explore such patterning of COs, we computed the distribution of inter-CO distances (ICDs)—the separation (in bp) between successive COs along every chromosome (Fig. 2A). To accommodate comparisons between samples with different numbers of COs, ICD lengths were transformed by dividing by the mean ICD size (see the ‘Materials and methods’ section; Supplementary Fig. S1G and H) and visualized in rank order as empirical cumulative distribution functions (eCDFs; Fig. 2B–G). Transformation in this manner enables the distributions and variance in ICDs to be compared between datasets independently of any differences in CO frequency.

In wild-type and *msh2*∆ strains, CO distributions deviated significantly from simulated conditions in which the same frequency of observed COs were distributed randomly relative to one another (*P *<.01; two-sample KS test, Fig. 2A). In particular, observed patterns displayed a less variable distribution of ICDs than random, with fewer short and long distances between adjacent COs than expected by chance. This change in distribution was visible as a steeper eCDF curve in wild type experimental data compared to the random simulation, a feature that was stronger within the YJM × S96 hybrid (Supplementary Fig. S1J) than within SK1 × S288c (Fig. 2A), additionally suggesting that the meiotic CO landscape is modulated in a hybrid-specific manner (Fig. 2A and Supplementary Fig. S1J; see the ‘Discussion’ section).

Notably, inactivation of Msh2 in the SK1 × S288c hybrid caused the ICD curve to skew yet further away from the random simulation, creating a steeper inflection point (Fig. 2A; *P* <.01; two-sample KS test) indicative of decreased variance in the spacing of COs relative to one another. Importantly, such differences are unlikely to be explained by technical differences in the accuracy of calling CO positions, because simulations that randomly displaced CO midpoints by up to 5 kbp (Supplementary Fig. S2A), or randomly removed CO events (Supplementary Fig. S2B) did not introduce sufficient variation in the observed CO distributions to reconstitute the difference between wild-type and *msh2*∆ ICDs (Supplementary Fig. S2C–G). *MSH2* deletion in the YJM × S96 hybrid also caused a shift towards uniformity, albeit less strong than in the SK1 × S288c hybrid (Supplementary Fig. S1J, *P *= 0.285; two-sample KS test), indicating that the strength of the effect of Msh2 inactivation is variable between crosses.

### Differences in CO distributions are not caused by differences in CO frequency

To account for any impact increased CO frequency may have upon CO distribution, we analysed CO positions reported in an *ndt80AR* (“arrest–release”) strain where meiotic prophase length is extended via temporary repression of the Ndt80 transcription factor [52, 53], with meiotic cells induced to exit prophase after 8 h incubation in sporulation medium [39]. Such prophase extension allows more time for DSBs and resulting COs to form and therefore results in an increased number of COs [39]. On average, 95.4 ± 13.9 COs per meiosis were identified within *ndt80AR*—a significant increase relative to wild type (*P = *0.045, Wilcoxon test, Fig. 1B). No further significant increase occurred upon deletion of *MSH2* (*msh2*Δ *ndt80AR*, 98.3 ± 16.7 COs, *P *= 0.846, Fig. 1B). Density of COs across chromosomes were largely unchanged by *ndt80AR*, with the exception of a small elevation in subtelomeric CO density compared to wild-type and *msh2*∆ (Fig. 1E and F), a phenomenon described in more detail elsewhere [39]. Importantly, increased CO frequency alone (*ndt80AR*) did not alter CO distribution compared to wild type (Fig. 2B) (*P *= 0.764; two-sample KS test). Moreover, despite the lack of change in CO frequency, a near-identical *msh2*Δ-dependent skew in CO distribution was still observed in *ndt80AR msh2*∆ (Fig. 2C and D).

Inactivation of MMR within hybrid *S. cerevisiae* strains by deletion of *MSH2* therefore gives rise to two distinct phenotypes relative to wild type: (i) increased CO frequency, as previously observed, and (ii) a global shift in the distribution of COs relative to one another such that they are more evenly spaced—something that is independent of changes in CO frequency (though the shift is lesser in the S96 × YJM789 cross, see the ‘Discussion’ section). Although we are not directly measuring interference between COs by these analyses, the more evenly spaced CO distribution is consistent with the loss of MMR activity increasing the presence and/or impact of CO interference within the global pool of COs.

### Gamma distributions can estimate the global influence of interference

To quantitatively assess differences in CO distributions, we modelled CO patterns using gamma distributions, which have previously been used to model CO distributions in many systems [54–58]. Gamma distributions are described as two parameters: shape and scale, often referred to as alpha (α) and beta (β), respectively. Both α and β can be calculated from the mean and standard deviation of the fitted distribution [46]. Transforming data by dividing by the mean value only alters the scale (β) of the fitted gamma distribution, but it does not alter the shape (α). Furthermore, when mean = 1, α = 1/β. Transformed gamma distributions can therefore be described using shape alone.

A randomized population of sufficient size can be described by a gamma distribution with shape 1 [54]. Because the shape of a gamma distribution is inversely proportional to the population’s standard deviation, any distribution with less variance than that of a random distribution will therefore better fit a gamma distribution with a shape >1 [54]. Thus, after transformation, we can describe the uniformity of a population using the shape parameter of the best-fitting gamma, with more homogenous populations fitting gammas with larger shape parameters (Supplementary Fig. S1G–I). In the context of ICDs, a larger shape represents a more even distribution of COs, while a smaller shape corresponds to a more variable distribution of COs.

In the SK1 × S288c background, wild-type ICDs best fit a gamma distribution of shape 1.43 (Table 1). In contrast, *msh2*Δ ICDs best fit a gamma distribution of shape 2.41, consistent with the relatively lower variance in ICDs compared to wild type (Fig. 2B and Table 1). Notably, extension of meiotic prophase mediated by the *ndt80AR* allele appeared to have little effect on CO distributions, regardless of *MSH2* deletion (shape = 1.52 and 2.49 respectively; Fig. 2C and D and Table 1). Importantly, the changes in CO distributions caused by *MSH2* deletion are independent of the changes in CO numbers caused by the *ndt80AR*-dependent prophase extension. In the YJM × S96 background, wild-type ICDs best fit a gamma distribution of shape 1.85 while *msh2*∆ ICDs best fit a gamma distribution of shape 1.91, again suggesting a smaller effect of Msh2 in the YJM × S96 hybrid (Table 1).

**Table 1.** Summary statistics of whole-genome recombination data analysed in this study

Background	Genotype	Meioses	COs	NCOs	Events	CO shape	NCO shape	Event shape	CO ICDs	NCO ICDs	Event ICDs	CO *P*	NCO *P*	Event *P*
SK1xS288c	Wild type	6	75	31	105	1.43	1.20	1.36	59	18	90	<0.001	0.014	<0.001
SK1xS288c	*msh2*∆	13	106	92	199	2.41	1.21	1.50	90	101	183	<0.001	<0.001	<0.001
SK1xS288c	*ndt80AR*	8	95	47	143	1.52	0.93	1.17	79	32	127	<0.001	0.366	0.001
SK1xS288c	*msh2*∆ *ndt80AR*	6	98	85	184	2.49	1.22	1.34	82	70	168	<0.001	<0.001	<0.001
SK1xS288c	*zip3*∆	4	47	86	133	1.15	1.24	1.27	38	70	117	0.246	<0.001	<0.001
SK1xS288c	*zip3*∆ *msh2*∆	4	26	339	365	1.00	1.46	1.49	18	323	350	0.977	<0.001	<0.001
YJMxS96	Wild type	52	92	47	139	1.85	0.75	1.20	76	32	123	<0.001	<0.001	<0.001
YJMxS96	*msh2*∆	4	117	56	173	1.91	1.22	1.44	101	42	162	<0.001	0.013	<0.001
YJMxS96	*zip3*∆	7	61	122	183	1.13	1.37	1.37	46	106	173	0.173	<0.001	<0.001

Background: Diploid hybrid background. Genotype: Relevant genotype for comparison. Meioses: The number of four-spore viable tetrads (or eight-spore viable octads) analysed.

COs: Average number of COs per meiosis.

NCOs: Average number of noncrossovers per meiosis.

Events: Average number of recombination events (CO + NCO) per meiosis.

CO shape: Shape of best single gamma model fitted to ICDs (see the ‘Materials and methods’ section).

NCO shape: Shape of best single gamma model fitted to INCDs (see the ‘Materials and methods’ section).

Event shape: Shape of best single gamma model fitted to IEDs (see the ‘Materials and methods’ section).

ICDs: The average number of ICDs recorded per meiosis.

INCDs: The average number of INCDs recorded per meiosis.

IEDs: The average number of inter-event (CO + NCO) distances recorded per meiosis.

CO P: Two-sample KS test *P*-value between ICDs and best-fitting single gamma model (shape parameter noted in CO shape).

NCO P: Two-sample KS test *P*-value between INCDs 20and best-fitting gamma model (shape parameter noted in NCO shape).

Event P: Two-sample KS test *P*-value between IEDs and best-fitting gamma model (shape parameter noted in Event shape).

Because *ndt80AR* itself does not have a significant effect on ICD distributions in either the presence (Fig. 2C; “MMR+”) or absence (Fig. 2D; “MMR−”) of *MSH2*, genotypes ± Ndt80AR were grouped by *MSH2* status and considered as a single population for the purposes of the following analyses in order to decrease sampling error and thus to increase statistical power. MMR+ CO distributions (wild type and *ndt80AR* combined) are fitted best by a gamma distribution of shape 1.48 (Fig. 2F) while the MMR− CO distributions (*msh2*∆ and *msh2*∆ *ndt80AR*) instead fit a gamma distribution of shape 2.44 (Fig. 2G), and are thus much more uniform.

The observed differences in ICD distributions are potentially indicative of differences in the influence of CO interference. To directly assess CO interference, we employed Coefficient of Coincidence (CoC) analysis, an accepted measure of interference strength (Fig. 2A) [20]. In order to increase the statistical power of this analysis, ICDs were pooled across chromosomes prior to CoC calculation (see the ‘Materials and methods’ section). In CoC analysis, values lower than one at a given distance are indicative of suppression of CO coincidence i.e. indicative of positive interference. Wild type and *msh2*∆ CoC curves differed, with the *msh2*∆ CoC value starting lower and increasing to ∼1 more slowly than wild type (Fig. 2H). Similar differences in CoC were observed when comparing *ndt80AR* with *ndt80AR msh2*∆ (Fig. 2I and J) suggesting, as in the ICD analysis, that *MSH2* deletion has the same effect in both *NDT80* and *ndt80AR* cells (Fig. 2K). Indeed, grouping cells by *MSH2* status, as was done for ICD distributions to increase statistical power, reinforced this Msh2-dependent difference (Fig. 2L), which indicates that COs display more interference between one another in the absence of Msh2. L_CoC_, a metric that describes the distance at which CoC = 0.5 [20], is <10 kb in MMR+ strains and ∼36 kb in MMR− strains (Fig. 2L).

### Simulations suggest increased proportions of class I COs upon *MSH2* deletion

We considered two explanations for the observed differences in CO distributions and interference ± *MSH2*. The first possibility is that interference changes, either in strength and/or in range, with stronger and/or greater ranging interference producing a more uniform distribution of COs upon *MSH2* deletion. The second possibility is that the proportion of COs that contributes to the interfering pattern is altered. From a biological perspective, because both class I and class II COs exist in the dataset, but are otherwise indistinguishable, any increase in the ratio of class I:class II COs is expected to result in a more uniform CO distribution and a decrease in CoC values at short distances.

An earlier iteration of this study investigated the use of maximum-likelihood estimation (MLE) to fit mixtures of two independent gammas [59]. However, such models have a number of limitations. First, gamma models predict ICDs that are impossible in the experimental data due to truncation by chromosome space and event merging (Supplementary Fig. S3A). Second, predictions made by mixed gamma models become inaccurate when applied to relatively pure mixtures of CO types (Supplementary Fig. S3B and C). Finally, most importantly, interfering and noninterfering COs exist in the same genomic space, and therefore mixtures are not independent (Supplementary Fig. S3D).

**Figure 3.**
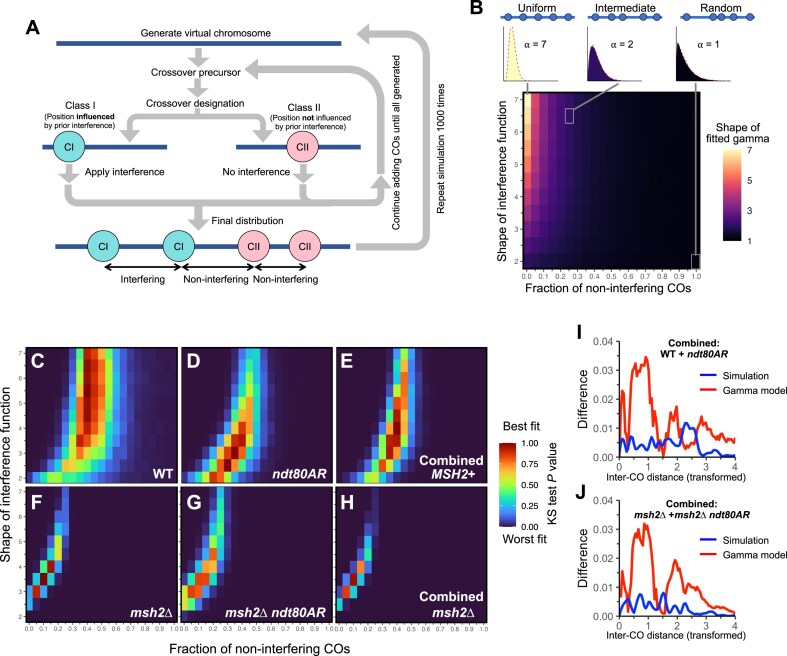
Wild type and *msh2*∆ ICD distributions vary due to different fractions of interfering COs and not due to differing interference strength. (**A**) Schematic of simulation strategy used to generate synthetic ICD distributions. (**B**) Coloured heatmap of the shape (⍺) of gamma distributions fitted to simulated ICD distributions. Each pixel represents a particular combination of parameter values: the shape of the interference function (Y-axis) for the interfering proportion of COs, with the proportion of noninterfering COs varying on the X-axis. Smaller plots are probability density functions of example ICD distributions with representative fits, with parameters matching indicated pixels. Red dotted lines represent gamma models fitted to simulated data. (**C**–**H**) Coloured heat maps of *P*-values (two-sample KS test) between observed and simulated CO distributions expressed as eCDF curves for the indicated strains and/or combined datasets. *P*-values >.9 indicate good statistical fits. Each pixel represents a particular combination of parameter values as presented in panel (**B**). See main text and the ‘Materials and methods’ section for more details. (**I, J**) Simulations have better fits than single gamma models. Difference in cumulative density functions between experimental data in the indicated combined datasets and indicated models of ICDs; simulated data (blue) and fitted single gamma model (red).

In an attempt to more accurately model CO distributions, a dedicated simulator was employed to generate artificial COs using a range of parameters (see the ‘Materials and methods’ section, Fig. 3A, and Supplementary Fig. S4). In brief, the simulator iteratively places COs along sixteen separate chromosomes, modeling the *S. cerevisiae* genomic space and applying repressive “interference” around events designated as class I COs, but not those designated as class II COs. Subsequent positioning of class I COs is influenced (repressed) by such interference, but class II positioning is not (Fig. 3A).

To estimate the fraction of class II COs in wild-type data, the CO simulator was run independently 1000 times, each simulation halting when the total number of COs generated reached the same number as the average observed in the wild-type experimental data (e.g. *N* = 75). Simulations were then repeated while varying the probability of class I/class II designation between runs (0–1). Separately, the strength of the interference function (as determined by the shape parameter) was also varied between shapes of 2 and 7, thereby testing a range of increasing interference strengths. To assess the overall effect that mixtures have on the combined ICD distributions, each (mixed) simulation was fitted with a single gamma function and the results presented as a heat map (Fig. 3B).

As expected, the overall shape parameter of models fitted to simulated data was greatest when the interference shape parameter was largest and when noninterfering COs were absent (Fig. 3B). Notably, however, introduction of even small proportions of noninterfering COs had a substantial effect on the shape of mixed CO distributions, which come close to a shape of 1 before the class II proportion reaches 0.5 no matter how strong the interference generated around class I COs was (Fig. 3B). This effect is likely due to the introduction of not just noninterfering class II–class II ICDs, but also class I–class II ICDs, which also display a random, noninterfering distribution (Fig. 3A and Supplementary Fig. S3D).

Next, the simulated mixed CO distributions were compared to wild-type data using a two-sample KS test in order to determine which of the simulated distributions best matched the experimental data. Best fits for wild-type data arose when the random (class II) proportion was between 0.35 and 0.45 (Fig. 3C). A relatively wide range of well-fitting shape parameters for the interfering fraction were observed (3–7), consistent with a distribution of high standard deviation. These observations suggest that 35%–45% of all COs are noninterfering (class II) COs in wild type *S. cerevisiae*, with the remaining (class I) COs displaying a distribution that is fit with a relatively strong interference pattern (shape >3).

The above analysis was then repeated with *ndt80AR* data. Surprisingly, despite the previously noted statistical similarity between wild-type and *ndt80AR* CO distributions, the best-fitting simulations for *ndt80AR* data were slightly different (30%–40% class II, interference shape 2.5–4, Fig. 3D). This relatively smaller range of good fits likely reflects the higher number of COs in the *ndt80AR* dataset, and the resulting increase in statistical power. The pooled MMR+ CO distribution best fitted a range of 35%–40% class II COs, with the class I interference shape 3–4—parameter ranges that also overlapped good fits of both wild-type and *ndt80AR* CO distributions (Fig. 3E).

We next repeated the above analysis to identify simulations that matched *msh2*∆ data (Fig. 3F–H). In contrast to the *MSH2* wild-type analyses above, best fits arose when the class II proportion was 5%–15%—much lower than observed in wild-type cells—yet where the shape of the interference function remained similar (between 3 and 4) (Fig. 3F). These same optimal values were found when matching simulations to either *msh2*∆, *msh2*∆ *ndt80AR* or the pooled MMR− (*msh2*∆ + *msh2*∆ *ndt80AR*) datasets (Fig. 3G and H).

Differences between wild type and *ndt80AR* were not observed in previous analyses (Fig. 2C), but are suggested to be identified due to the increased sensitivity of the simulator. Notably, the simulator detected no obvious difference ± *ndt80AR* in the *msh2*∆ background. Taken together, these results suggest there is a small Msh2-dependent difference in *ndt80AR*, but that this effect is far weaker than the strong effect that arises upon deletion of *MSH2*, which is observable in both the wild-type and the *ndt80AR* backgrounds. Thus, the effect of *MSH2* deletion is most simply explained by an increase in the fraction of interfering (class I) COs, without any substantial change in interference strength (shape) itself. Overall, simulations themselves produced better fits for both wild-type and *msh2*∆ ICD distributions than single-gamma models (Fig. 3I and J) further supporting the possibility that two distributions of ICD are present in the data.

Recently, it was proposed that all COs display interference, but that two types of CO exist which do not display interference between one another [60], henceforth referred to as type A and type B (Supplementary Fig. S5A). To test this concept, the simulator was modified to employ two independent patterns of interference—one for each type of CO—with both the strength of type B interference and the fraction of type B COs altered across different simulation runs, whilst fixing the type A interference shape as 2 or 3 (Supplementary Fig. S5B and C). Such simulations revealed a number of potential good fits for wild-type and *msh2*∆ data, suggesting that such a model could fit the observed CO distributions (Supplementary Fig. S5C–H). Nevertheless, whether this model is correct or not, these simulations again suggested that COs in MMR− genotypes are predominantly composed of a single type, whilst MMR+ COs are a more even mix of the two types (Supplementary Fig. S5C–H).

Inefficient CO maturation—downstream of the implementation and patterning effects of CO interference—has also been proposed as a mechanism to explain CO patterns and the innate predisposition towards chromosome missegregation in human females [21]. To investigate the possibility of differential rates of CO maturation failure explaining the effects of Msh2 on CO distributions, we modelled failed COs as “invisible” events that generate interference (and therefore impact the placement of subsequent COs) but are not counted as COs for the purposes of ICD calculation (Supplementary Fig. S6A). Such a model emulates a situation in which COs fail after generating interference. Though increasing the proportion of failed COs increased the amount of randomness observed in the ICD distributions (i.e they are best fit with lower shape values; Supplementary Fig. S6B), maturation failure does not appear to have a sufficiently large effect on CO distributions to account for all of the differences observed ± Msh2 (Supplementary Fig. S6C–H).

Thus, collectively from these analyses, we conclude that both the absolute number of total COs and the proportion of those COs that display interference (class I COs) are increased when *MSH2* is deleted.

### Cytological examination of class I COs supports that Msh2 does not affect CO interference

**Figure 4.**
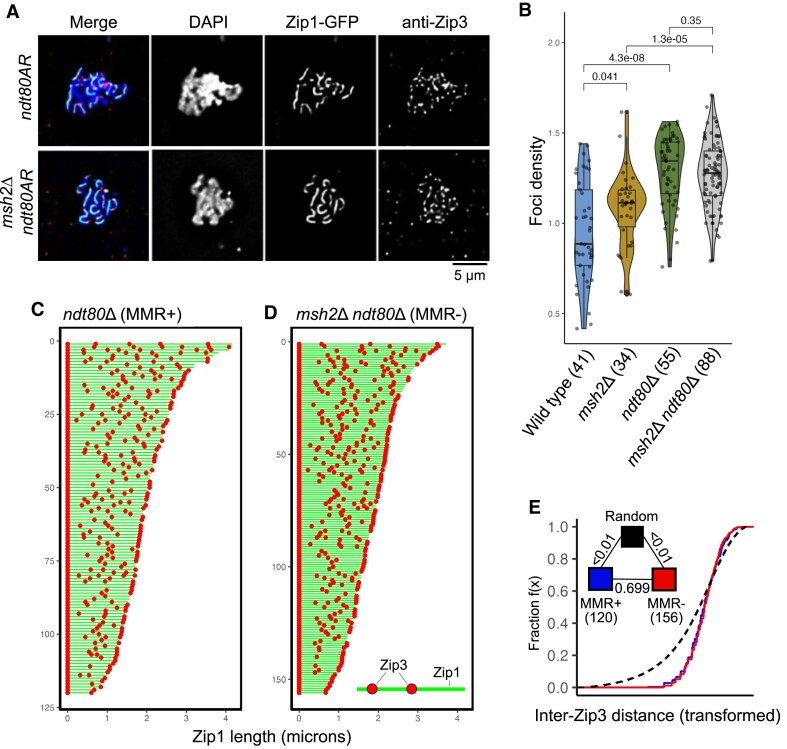
Distances between Zip3 foci along synapsed chromosome axes are not different ± *MSH2* suggesting interference strength does not change. (**A**) Representative images for each genotype. Chromosome spreads were fluorescently labelled for the meiosis-specific synaptonemal complex protein Zip1-GFP (green), the class I CO marker, Zip3 (red) and DNA (DAPI, blue). Only well-spread nuclei with clear Zip1 threads were analysed. Only Zip3 foci overlapping within the DAPI-stained area were counted. (**B**) Box-and-whisker plot showing Zip3 foci density from pachytene chromosome spreads of S288c × SK1 hybrids. Midlines denote median values, box limits are first and third quartile, whiskers are highest/lowest values within 1.5-fold of interquartile range. The total number of nuclei counted is indicated in brackets obtained from three independent experiments. *P*-values: Two-sample Wilcoxon test. (**C, D**) Relative distribution of Zip3 foci along individual Zip1-GFP positive chromosome axes in *ndt80AR* (**C**) and *msh2*Δ *ndt80AR* (**D**) in pachytene-arrested cells (8 h after meiosis induction), ordered from top to bottom by decreasing axis length (Green bar, measured Zip1 axis length; red dot Zip3 focus position). (**E**) eCDFs showing the fraction of inter-foci distances at or below a given size. Distances were transformed by dividing by the mean ICD length (see the ‘Materials and methods’ section) to correct for skews generated by differing total foci frequencies. Black dashed lines represent a randomized distribution generated via simulation to represent a state of nonuniformity (see the ‘Materials and methods’ section). Pairwise GoF tests were performed between genotypes as indicated (triangular legend). *P*-values: Two-sample KS test.

Zip3 foci are thought to mark the sites of interfering, class I COs on pachytene synapsed chromosomes, but not noninterfering class II COs [20, 61]. Given that our analysis predicts that there are greater numbers of class I COs in *msh2*∆ than in wild type without any substantial difference in the strength of interference between those COs, we would expect to see an elevated number of Zip3 foci in the absence of Msh2, but no change in the distribution pattern of Zip3 foci. Thus, to test the results of the simulator analysis, we counted Zip3 foci [4] on spread meiotic chromosome preparations from S288c × SK1 hybrids co-labelled with Zip1-GFP, an established marker of chromosome synapsis in *S. cerevisiae* (Fig. 4A) [62, 63]. To reduce observational bias, samples were randomized, and counting was restricted to only well-spread nuclei showing clear thread-like patterns of Zip1.

Overall, focus number per nucleus was highly variable (10–50 per cell; Supplementary Fig. S7A). Perhaps because of this variation, no reproducible differences in foci number upon *MSH2* deletion could be identified, in neither the wild type nor the pachytene-arrested *ndt80*∆ strain background (Supplementary Fig. S7A). However, the possibility of this high variance obscuring a real difference cannot be discounted. Within these data we noticed a modest correlation between spread size and total foci count per spread (Supplementary Fig. S7B). Thus, to investigate whether technical differences in spreading efficiency was affecting Zip3 foci counts we further analysed the density of Zip3 foci per µm^2^ of spread area bounded by the chromosomal (DAPI) signal (Fig. 4B). Notably, we observed a modest increase in foci density in *msh2*∆ compared to wild-type (*P *= 0.041, two sample Wilcoxon test, Fig. 4B). Foci density was also significantly increased in *ndt80*∆ (*P = *4.30 × 10^−8^, Two sample Wilcoxon test, Fig. 4B), but no further increase was seen in *msh2*∆ *ndt80*∆ (*P *= 0.35, two sample Wilcoxon test, Fig. 4B). Together, these analyses suggest that variations in spreading may be obscuring real differences in foci number between wild type, *msh2*∆, and *ndt80*∆ cells.

Although we cannot confidently conclude that there are differences in Zip3 foci number, we considered that any real difference in interference between class I COs might be detectable as changes in the relative distributions of Zip3 foci when assessed along synapsed chromosome axes. To test this we analysed such distributions in cells arrested at the pachytene stage via the *ndt80*∆ allele (Fig. 4C and D). Measuring the distances between Zip3 foci along the subset of well-resolved chromosomes in each strain demonstrated significant deviation from that expected for a random distribution (*P *<.001, two-sample KS test, Fig. 4E). Inter-Zip3 distances best-fitting gamma distributions of shape 4.96 (*ndt80*∆) and 5.69 (*msh2*∆ *ndt80*∆), indicative of highly interfering distributions—as expected for Zip3 foci marking interfering class I events [20]—that were not significantly different in the absence of Msh2 (*P *= 0.699, two-sample KS test, Fig. 4E), again suggesting that the strength of interference has not changed.

Whilst the simulated alpha for class I COs was ∼3–4, substantially lower than that measured by microscopy (∼5–6), this difference is likely explained by opposing biases in the two assays. Although CO positions measured from sequencing data have relatively high precision it is accepted that CO patterning acts along chromosome axes rather than DNA basepairs [64, 65]. In this context, nonuniform rates of DNA packing along chromosome lengths [66] will generate skews that cause alpha/shape values to be underestimated. By contrast, microscopic identification of Zip3 foci has constrained spatial resolution for detecting the shortest of distances between adjacent COs—a skew that will cause an overestimate in alpha/shape.

It is important to note that, since Zip3 may be present prior to pachytene, Zip3 may be marking more than class I COs [67]. However, if a substantial number of events other than class I COs were counted, we would expect our assay to suggest class I COs are less evenly spaced than they truly are. Thus, we can overall conclude that whilst Msh2 appears to affect the number of successful class I COs, it does not affect the spatial distribution between those COs. Because Msh2 can act upon recombination intermediates containing mismatches, we hypothesize that the effects we observe are caused by the potentially selective action of Msh2 upon the precursors of class I designated events.

### Msh2 suppresses class I COs in regions of high polymorphism density

**Figure 5.**
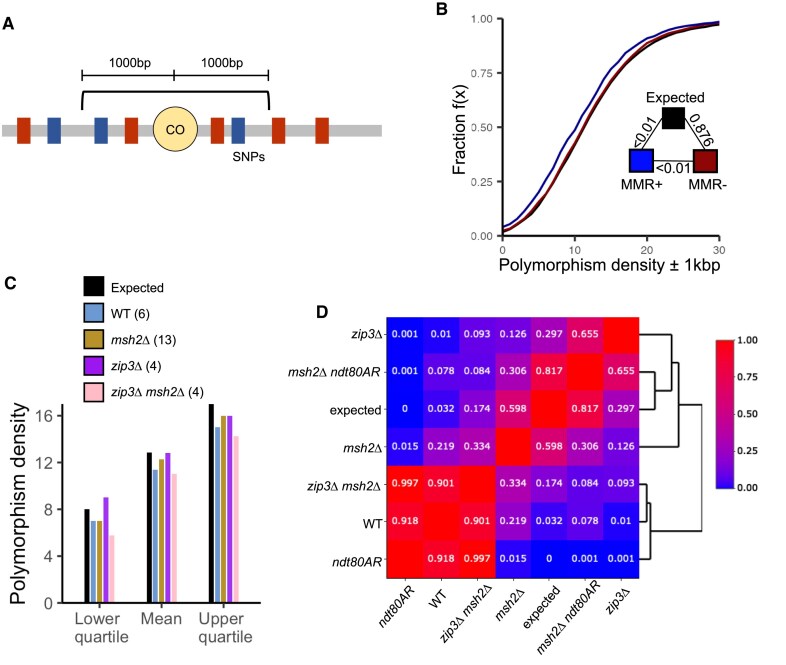
Bias of COs towards regions of lower polymorphism density depends on Msh2. (**A**) Polymorphisms within 1 kb of events were counted to calculate polymorphism density. All SNPs and indels were weighted equally. (**B**) eCDFs showing the fraction of COs that reside within a region of a given polymorphism count in S288c × SK1 MMR proficient (wild type, *ndt80AR*) compared to MMR deficient (*msh2*∆, *msh2*∆ *ndt80AR*) strains. Black curve is the expected association based upon polymorphisms around Spo11-DSB hotspots [48]. (**C**) Mean, lower and upper quartile polymorphism densities of COs in the indicated strains. (**D**) Similarity of polymorphism densities in the indicated strains. Colours and numbers within each cell represent the two-sample KS test *P*-value between indicated strains.

The MMR machinery forms a potent barrier to homologous recombination, presumably due to recognition and destabilization of recombination intermediates containing DNA mismatches [49–51]. Given the Msh2-dependent influence on CO distribution reported above, we next investigated the interplay between DNA mismatches and CO formation, by calculating polymorphism densities (SNPs, indels) ± 1000 bp around every mapped CO (Fig. 5A) and comparing between genotypes. To generate a comparative reference point, the expected environment for meiotic recombination, as defined by the polymorphism density surrounding ∼3600 DSB hotspot midpoints [48], was also calculated.

Polymorphism density surrounding COs within *MSH2* wild-type strains (S288c × SK1: wild type, *ndt80AR*) was significantly different to that expected in both distribution (*P *<.001; two-sample KS test; Fig. 5B) and in mean variant density (11.4 versus 12.5, *P *= 0.002; two-sample Wilcoxon test; Fig. 5C) characterized by a skew towards COs, on average, arising within regions of lower genetic divergence than expected from the genome-wide position of DSBs. By contrast, the polymorphism density around *msh2*Δ COs displayed statistical similarity to that expected (*P *= 0.876; two-sample KS test; 12.5 versus 12.9, *P* = 0.538; two-sample Wilcoxon test, Fig. 5B). Such a disparity between wild type and *msh2*Δ was recaptured within the independent S96 × YJM789 hybrid cross, where COs were again skewed towards regions of lower polymorphism density only in the *MSH2* wild-type strain (Supplementary Fig. S8A–C). This effect, in both hybrid backgrounds, was diminished with increasing distance (±2000 bp; Supplementary Fig. S8D–I), suggesting that DNA mismatches exert a localized inhibitory effect on CO formation. In contrast to COs, NCOs in *msh2*∆ strains appear skewed towards higher polymorphism densities than expected, while in *MSH2* strains, NCOs closely resemble the expected distribution (Supplementary Fig. S8J–L). Importantly, however, despite this apparent difference in behaviour, noncrossovers require overlapping polymorphisms for their detection, thus we are cautious of making overly strong conclusions from this difference.

Given that our simulations suggest that Msh2 activity suppresses class I CO abundance, we hypothesized that Msh2-dependent effects may be limited to suppression of class I COs. To investigate this possibility, we assessed polymorphism density in *ZIP3* mutants (Fig. 5C). Notably, COs in the *zip3*∆ strain showed a similar relationship to polymorphism density as did COs in *msh2*∆ (no skew towards less-polymorphic regions), suggesting that class II COs are not subject to polymorphism density-dependent inhibition even when Msh2 is present (Fig. 5C).

Surprisingly, however, COs in the *zip3*∆ *msh2*∆ strain exhibited the opposite effect—restoration to a skew away from higher polymorphic regions similar to that observed in wild type (Fig. 5C and D). It should be noted, however, that the difference in polymorphism densities around COs between *zip3*∆ and *zip3*∆ *msh2*∆ was only borderline significant (*P* = 0.093, two-sample KS test), likely due to the low numbers of COs observed in the latter strain. Nevertheless, such skew could potentially indicate that Msh2 stabilizes class II COs and/or their precursors, though this effect is only detectable in the absence of the class I CO pathway.

### Effects of Msh2 on class I COs are Zip3 dependent

Our results above suggest that Msh2 may act on both classes of CO: discouraging the formation of class I COs in areas of high polymorphism density whilst potentially having the opposite effect on class II COs. In order to study the effects of Msh2 on class II COs, we assessed CO numbers and distributions in a further two strains—a *zip3*∆ strain and a *zip3*∆ *msh2*∆ strain.

**Figure 6.**
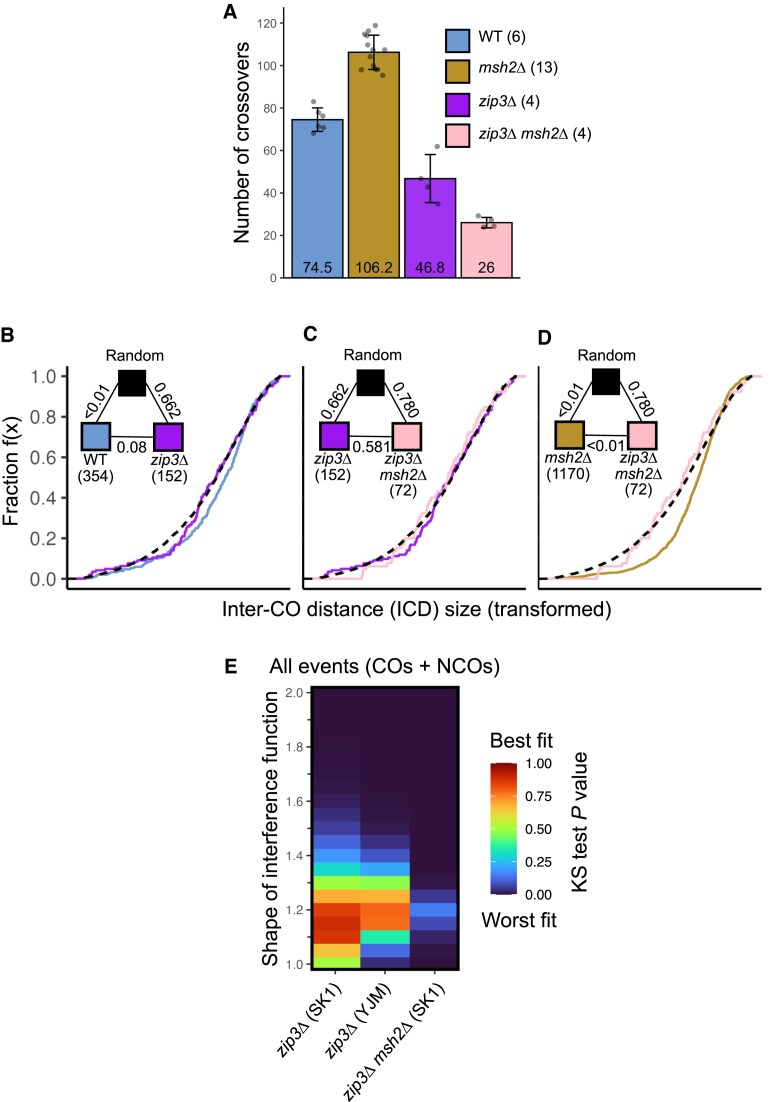
The effects of Msh2 on COs is dependent on Zip3. (**A**) Individual (grey circles) and average (bars and inset numbers) number of COs per meioses for each genotype. The number of individual meioses sequenced per genotype is indicated in brackets. Error bars represent standard deviation. (**B**–**D**) eCDFs showing the fraction of ICDs at or below a given size. ICDs were transformed by dividing by the mean ICD length (see the ‘Materials and methods’ section) to correct for skews generated by differing CO frequencies. Black dashed lines are datasets generated via simulation to represent a random distribution (see the ‘Materials and methods’ section). Pairwise GoF tests were performed between genotypes as indicated (triangular legend). *P*-values: Two-sample KS test. Numbers in brackets indicate the total number of ICDs in each genotype. (**E**) Coloured heat maps of *P*-values (two-sample KS test) between observed and simulated CO + NCO distributions expressed as eCDF curves for the indicated strains. *P*-values >.9 indicate good statistical fits.

Consistent with the loss of the class I pathway, deletion of *ZIP3* caused a significant decrease in the number of COs (46.8 COs, Fig. 6A, *P* = 0.014, two-sample Wilcoxon test) and an increase in the number of noncrossovers (86 noncrossovers; Supplementary Fig. S9A). Effects of a similar magnitude were observed in the YJM × S96 hybrid (Supplementary Fig. S9B and C). Interestingly, in contrast to the wild-type background, deletion of *msh2*∆ in the *zip3*∆ background caused a further decrease in CO number (26 COs, Fig. 6A, *P* = 0.029, two-sample Wilcoxon test), again potentially indicating that Msh2 promotes class II COs. A substantial increase in the number of noncrossovers was also observed (339; Supplementary Fig. S9A). Such a drastic increase in the number of visible noncrossovers is likely at least in part due to the lack of homologue engagement that arises when Zip3 is absent [68]. It is, however, important to note that the *zip3*∆ and *zip3*∆ *msh2*∆ strains had low viability (13.2 and 7.34% four-spore tetrads, respectively; Supplementary Table 2) [39]. Since our analysis relies on fully viable meiotic products, the phenotypes we observe here are biased towards those visible within four-spore-viable meioses and are potentially less extreme than the true effect.

Upon removal of the class I pathway, we would expect all COs to be class II and therefore exhibit no sign of interference. To test this, we compared *zip3*∆ ICDs to wild-type ICDs and a random distribution (Fig. 6B). As expected, *zip3*∆ ICDs were not significantly different to a random distribution (*P* = 0.662; two-sample KS test, Fig. 6B). The *zip3*∆ CDF curve is visibly shifted towards random relative to wild-type, though such a difference is only borderline significant (*P* = 0.079; two-sample KS test, Fig. 6B). Similar results were observed in the YJM × S96 cross, though wild-type and *zip3*∆ ICDs were significantly different, likely due to higher statistical power (*P *= 3.41 × 10^−5^; two-sample KS test; Supplementary Fig. S9D).

Deletion of *MSH2* in a *zip3*∆ background did not affect ICDs; ICD distributions in *zip3*∆ and *zip3*∆ *msh2*∆ were neither significantly different from each other (*P *= 0.581; two-sample KS test) nor the random distribution (*P = *0.662 and 0.780, respectively, two-sample KS test, Fig. 6C), confirming as expected that only noninterfering COs are present in both strains. The lack of both an increase in CO number and shift in ICD distribution away from random seen in *zip3*∆ *msh2*∆ suggests that the effects of *msh2*∆ are *ZIP3* dependent (Fig. 6D). Consistent with COs being randomly distributed in the absence of Zip3, ICDs in *ZIP3* mutants fit a wide range of highly variable simulations (Supplementary Fig. S9E and F).

Fitting of gamma models to *zip3*∆ and *zip3*∆ *msh2*∆ ICDs produces models with shape close to 1 (Table 1), again consistent with the absence of class I COs. Unexpectedly, inter-NCO distances better fit gammas with higher shape parameters, a phenomenon that is only seen in Zip3-deficient genotypes, in both crosses (Table 1). Moreover, remarkably, distances between all recombination events (CO + NCO) appeared to be more uniform than both the ICDs and the inter-NCO distances, again in Zip3-deficient genotypes only (Table 1). This unique property suggests that, in the absence of Zip3, COs and NCOs may arise from the same underlying distribution—and one that experiences a weak level of interference between each event.

However, as discussed previously, single gamma models have limitations due to the truncation of ICDs in chromosome space (Supplementary Fig. S3D). Thus to further characterize patterning between total events (CO + NCOs) in the *zip3*∆ strains, we ran event simulations that varied interference shape in the range of 1–2, and compared results to the IEDs observed in the *zip3*∆ strains (Fig. 6E). The *zip3*∆ IEDs best-fitting simulations with interference shapes of 1.1–1.2 in both the SK1 × S288c and YJM × S96 crosses, whereas the best fits for *zip3*∆ *msh2*∆ IEDs were in a slightly higher range (1.15–1.25), albeit with much lower *P*-values (Fig. 6E). Collectively, these results suggest that, in the absence of Zip3, there may be weak interference between COs and NCOs, and that the positioning of such events is not entirely random—perhaps caused by DSB-dependent interference [69, 70], or by nonrandom patterning upstream of DSB formation [20].

Taken together, our observations indicate that the repressive effects of Msh2 on both CO number and distribution are largely Zip3 dependent—and thus largely specific to the class I CO pathway.

## Discussion

### An interaction between sequence polymorphism and CO formation

Sequence divergence suppresses recombination within a wide range of eukaryotes including *S. cerevisiae, M. musculus*, and *H. sapiens* [31, 32, 36, 71–73]. Findings presented here expand upon these observations and suggest that the antirecombinogenic activity of Msh2, exerted at sites that contain mismatches, does not mediate an indiscriminate suppression of COs but rather acts disproportionately at sites of ZMM-dependent, interfering COs—thereby altering the spatial distribution of recombination across the genome by modulating the class I:class II balance (Fig. 5B). Our observations underscore how even the low rates of divergence (∼0.6%) present within intra-specific hybrids of *S. cerevisiae* can generate global changes in CO frequency, CO type, and genome-wide distribution. Nevertheless, in wild-type hybrids when Msh2 is active, COs still frequently arise within polymorphic homologous regions (Fig. 5B) and with class I COs still forming at a high rate (60%–65%; Fig. 3C and E). Mismatches do not, therefore, form an absolute barrier to class I COs, but as may be expected instead seem to influence the probability of their formation.

The precise mechanism of CO interference is unknown and remains the subject of much debate. Two major models have emerged that attempt to explain the formation of CO interference: the beam-film model [20, 74] and the coarsening model [75, 76]. Our simulator models interference as a decaying wave, and is therefore more similar to the beam-film model. However, because the end result of interference is a reduced probability of proximal CO formation, our results are compatible with either of the two models.

It is notable that our analyses utilize relative CO positions as reported in DNA space, whereas other models support units of chromosome axis length as the relevant measure of CO interference [20, 65, 77, 78]. This distinction may underpin the relative difference in uniformity of Zip3 foci (alpha ∼5–6) measured microscopically versus our inferred class I CO distributions (alpha ∼3–4) as measured in sequencing data. Such differences may arise either from the undercounting of closely spaced foci on spread chromosomes due to optical resolution limits, and/or from any nonuniformity in packing of DNA within loops along the chromosome axis, which may be prevalent even at pachytene (e.g. [66]). Whilst the relationship between chromosome loop structure and recombination outcome is as-yet unknown, it is interesting to consider that CO formation—and perhaps interference itself—might modulate local loop lengths, leading to more distally (and uniformly) separated COs as measured along axes than the DNA sequence distances would predict.

### Redirection of class I CO precursors

Because CO maturation failure has been proposed to potentially explain almost all human aneuploidies [21], it is important to consider at what stage Msh2 activity causes class I CO precursors to be redirected towards alternative outcomes. Prior analyses aimed at elucidating this process have harnessed relatively deep (rather than broad) datasets in order to build probability distributions of expected coincident Zip3-marked class I COs at adjacent positions along specific chromosomes [20]. By contrast, the genome-wide maps of CO position utilized in this study are broad, encompassing positional information for every CO on every chromosome in individual meioses, but are of limited depth at any given locus due to the limited throughput of genome-wide sequencing of meiotic progeny. Moreover, unlike the specificity of Zip3-focus analysis for class I COs, genome-wide CO maps are unable to distinguish between class I and class II COs at any given site. For these reasons, we have employed direct and indirect measures of interference strength: CoC and statistical analysis of ICD distributions, respectively. Both analyses indicate greater interference in the absence of Msh2, but such differences are weaker than may be expected, likely due to the inclusion of class II events which are expected to be invisible in analyses of Zip3 foci. Simulations enabled us to postulate that CO distributions differ in the absence of Msh2 due to different proportions of class II COs and not because interference strength changes, a distinction that analysis of neither CoC nor ICD distributions alone are able to make.

**Figure 7.**
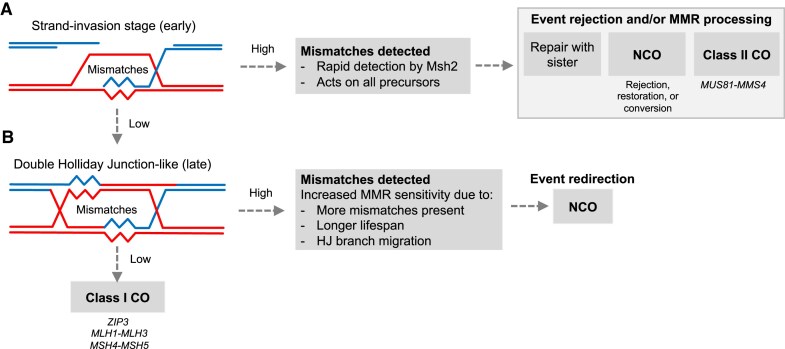
Model summarizing mismatch-directed suppression of class I COs and stabilization of class II COs. Mismatches (jagged lines) may arise within recombination intermediates at various stages of the meiotic recombination pathway due to differences in sequence between parental information (blue and red). (**A**) In the presence of a functional MMR pathway, regions of higher sequence divergence are proposed to give rise to transient heteroduplexes that cause Msh2-dependent redirection of repair toward the inter-sister, NCO or noninterfering (class II) CO outcomes. (**B**) Nascent recombination intermediates that survive initial heteroduplex recognition may continue maturing into the class I CO pathway, or more-rarely repair via the NCO or the noninterfering class II CO pathway. We propose that class I precursors become additionally sensitive to Msh2-dependent destabilization, perhaps due to intrinsic differences in structure, lifespan, and/or extent of heteroduplex DNA within them. For example, extended branch migration of Holliday junctions (HJs) at class I precursors [40] which may stabilize such intermediates [79] could increase the probability of hDNA arising within them, and thereby increase Msh2-dependent redirection away from class I CO outcomes. Thus, event redirection could arise via destabilization of nascent strand-invasion intermediates early (**A**), and/or via dissolution of double HJs via Sgs1–Rmi1–Top3 or disruption of CO-biased double HJ resolution (**B**).

When considering global CO positions, conversion from a class I to a class II CO would still give rise to a CO in the same position and with no change in global CO frequency. Thus, because we observe changes in CO pattern and frequency, we infer that at least some Msh2-redirected class I precursors become NCOs and/or become otherwise invisible within our assay (Supplementary Fig. S6F–H and S7). On this latter point, evidence exists for frequent repair-template switches between homologues and sister chromatids [40, 79], and thus redirection of CO precursors towards repair exclusively using identical sequences on the sister chromatid is also possible (events that would be invisible in our assays). However, such redirection would seemingly need to happen prior to the priming of DNA synthesis by a DSB end that has invaded the homologue (Fig. 7). Alternatively, Msh2-dependent redirection towards inter-sister events and NCOs may occur concomitantly with MMR, leading to restoration of any heteroduplex markers back to the parental configuration, again precluding detection by our methods (Fig. 7).

Thus, in our model (Fig. 7), rejected class I events may be redirected towards noncrossover outcomes relatively early on during CO designation and/or maturation via unwinding of the initial strand invasion intermediate and repair using synthesis-dependent strand annealing (SDSA). Alternatively, class I CO precursors that reach the double Holliday-junction (dHJ) stage could undergo Sgs1-Top3-Rmi1-dependent dissolution, an outcome perhaps stimulated by the potential for dHJs to undergo branch migration [40, 79], thereby generating large patches of heteroduplex DNA that could be detected by the MMR machinery.

Exactly how MMR-specificity for class I COs arises remains unclear. Msh2, Mlh1 and Pms1 form a ternary complex during MMR, and *in vitro* data suggest that Mlh1–Mlh3—essential class I CO factors—facilitate binding of Msh2 to heteroduplex DNA arising, for example, at sites where mismatches exist between parental strains [80]. Mlh1–Mlh3 may therefore be responsible for the differential sensitivity of each CO subclass to sequence mismatch through preferential recruitment or activation of Msh2 and MMR at class I sites. Indeed, available data [81] suggest that class I COs are more likely to recruit MMR and lead to conversion/restoration (6:2, 4:4 patterns)—or possibly rejection of the event (our results). By contrast, class II COs may be less likely to recruit MMR, thus surviving in MMR-proficient cells and thus more likely to show signs of post-meiotic segregation [81]. Such a model would fit with specific reductions in class I COs in MMR-proficient cells that we observe.

Our apparent inability to detect Msh2-dependent differences in the frequency of Zip3-marked CO precursors even at the pachytene-like arrest enforced by *NDT80* deletion where dHJs accumulate [82] indeed suggests that at least some class I redirection may arise after this dHJ stage. A late-stage activity of Msh2 in impeding class I CO formation would also be partially compatible with our simulations of CO maturation failure in wild-type but not *msh2*∆ cells. However, such effects of Msh2 appeared cross specific, for reasons that are as-yet unclear (Supplementary Fig. S6C–J). In the SK1 × S288c cross, Msh2 altered the estimated fraction of class II COs, while in the YJM × S96 cross, Msh2 instead increased the probability of class I CO failure. Though these results may seem contradictory, both can be explained by Msh2 acting to induce failure of class I COs—the difference is whether Msh2 acts on class I CO precursors before, during, or after interference is generated. S288c × SK1 yeast hybrids are reported to be partially MMR deficient [83]. However, even if so, our results favour the interpretation that MMR is in fact more deficient in the YJM × S96 hybrid given that *MSH2* deletion has a weaker effect on ICD distributions in the YJM × S96 hybrid (Fig. 1C and Supplementary Fig. S1J). Alternatively, it may be that the higher efficiency and well-synchronized progression of SK1 through meiosis [83] alters when Msh2 exerts the apparent inhibition of some class I COs.

Notably, CO distributions in the absence of Zip3 cannot be confidently distinguished from random distributions. However, gamma models fitted to *zip3*∆ *msh2*∆ recombination event distances (CO + NCO) have a shape >1, suggesting lower variance than random and thus greater uniformity. Such a skew from randomness could be due to detecting the contribution of DSB interference, which is believed to occur upstream of CO formation [69, 84, 85]. However, because NCOs are difficult to detect in our assay, we hesitate to declare this to be the true measure of DSB interference, which could be stronger or weaker than gamma models suggest. Furthermore, as with CO interference, the effect of DSB interference are likely to be influenced by local axis length and conformation rather than solely DNA length [69, 84, 86]. Moreover, as noted previously, nonuniform DNA packing within adjacent loops [66] is likely to blur any analyses that use DNA space.

It is also possible that the distributional differences in CO patterns we have observed are patterned by processes that are independent of CO interference and the class I or class II CO pathways. Nonuniform densities of DNA-sequence polymorphisms, DSBs, and even COs themselves all have the potential to influence the relative CO distributions that arise on a per-cell basis. However, polymorphisms, DSBs, and COs are relatively evenly spread across each chromosome in *S. cerevisiae* (Fig. 1C–F and Supplementary Fig. S10A–D), and thus, as expected, biasing CO site selection by these underlying population-level parameters had little impact on resulting patterns of simulated inter-CO distributions (Supplementary Fig. S10E–G). Nevertheless, we recognize that in organisms with less uniform SNP/indel density, and/or propensity to initiate recombination, such nonuniformity could result in a redistribution of CO formation towards certain regions, potentially influencing relative CO patterning on a per-cell basis (e.g. the effect that heterologous regions have in *A. thaliana*) [87].

In mitotic cells, inhibition of homologous recombination by means of heteroduplex rejection, relies upon Msh2 and the RecQ-family helicase, Sgs1 [50, 88]. An *sgs1*Δ mutant may therefore be expected to phenocopy *msh2*Δ if suppression of class I COs occurs via this mechanism. Intriguingly, however, the distribution of COs is more random in *sgs1*Δ relative to both wild type (Supplementary Fig. S11A) and *msh2*∆ (Supplementary Fig. S11B), consistent with a decrease in the proportion of class I COs. Moreover, *sgs1*∆ also abolishes the increased skew towards uniformity (inferred above to indicate an increased frequency of class I COs) caused by *MSH2* deletion (Supplementary Fig. S11C)—suggesting that Msh2 and Sgs1 are not epistatic, but rather antagonistic in the formation of class I COs. Notably, combined depletion of *SGS1* and *MSH2* in meiosis restores fertility in hybrids of two different yeast species [36]. Thus, putting this information together suggests that Msh2 mediates suppression of class I COs in a pathway different to that of Sgs1-mediated heteroduplex rejection, instead relying upon the downstream properties or factors of MMR, including Pms1 (Supplementary Fig. S11D), to achieve its effect. Alternatively, the genetic complexity outlined above may arise because Sgs1 can act at multiple steps and on a range of recombination intermediates [11, 89, 90]. For example, at early stages Sgs1 could act to promote class I CO formation by unwinding nascent recombination intermediates—independently of Msh2 and mismatches—thereby allowing them to be recycled into future potential class I precursors [91, 92]. By contrast, perhaps mediated by mismatch- and Msh2-dependent destabilization of pro-class I CO factors, Sgs1 activity at a later stage could promote dHJ dissolution thereby suppressing class I maturation.

### Possible divergence in Msh2 function between species

Looking outside of the fungal kingdom, Msh2 has been reported to promote (rather than suppress) the formation of interfering COs in regions of high polymorphism density in *A. thaliana*, in contrast to the results presented here [93]. Such a difference could be due to orthologue expansion in plant *MSH2*, allowing for divergence in function [94]. There are also major differences in genome structure between *A. thaliana* and *S. cerevisiae*, which could explain why the effects of Msh2 differ between these species. *Arabidopsis thaliana* has a much larger genome than *S. cerevisiae* (135 versus 12 Mb) [95, 96], which is also much more gene sparse, and has a much higher proportion of noncoding sequences (90% versus 25%) [97, 98]. However, *A. thaliana* genomes appear to have significantly less linkage disequilibrium (LD) than would be expected in such a large genome [99, 100], especially since COs are much less frequent than in *S. cerevisiae* [101–103]. Msh2 could be partly responsible for such a phenomenon, directing COs to highly polymorphic regions and causing recombination events to more efficiently break LD between SNPs, enhancing the effects of recombination.

Alternatively, the apparently contrasting effects of Msh2 in *A. thaliana* and *S. cerevisiae* may actually be the result of a shared function. For example, like in *S. cerevisiae*, COs in *A. thaliana* are significantly less common in regions of high divergence than in those regions with low divergence [104, 105]. Notably, contrasting the relatively even spread of polymorphisms across the *S. cerevisiae* genome (Supplementary Fig. S10A–D), regions of high and low sequence divergence exist in *A. thaliana* [87]. As such, it is possible that Msh2 could be inhibiting COs in divergent regions in *A. thaliana*, similar to the outcome in wild-type *S. cerevisiae* (Fig. 5B). However, if no COs form, it is possible that DSB formation is prolonged (due to a lack of local homologue engagement) [68, 106], causing DSBs to become more frequent in such regions relative to the rest of the genome. As a result, CO may eventually form (perhaps in sub-regions with few polymorphisms) simply because there are so many CO precursors. In such a model, COs would then appear to increase in frequency in polymorphic regions, as an indirect consequence of prolonged Msh2-dependent (and therefore mismatch-dependent) CO suppression.

Finally, as previously discussed, CO interference is proposed to work by a process of coarsening in *A. thaliana* [75, 76], but via localized inhibition more akin to formation and relief of stress in *S. cerevisiae* [20, 74]. Such differences may therefore indicate a difference in the hierarchy of CO designation and/or interference that has at least one additional layer of regulation in plants, and possibly also in other organisms with relatively large chromosomes. Perhaps, even if COs are suppressed at fine scale by sequence divergence in both organisms, higher-order biasing, and/or maturation of CO positions in plants via coarsening leads to a general co-association of COs with regions that are more polymorphic via mechanisms that are as yet undetermined.

### Outlook

Understanding the molecular mechanisms that contribute to speciation is fundamental to our understanding of biological diversity and evolution. Because class I CO formation is important for correct meiosis I chromosome segregation, our observations provide a mechanistic insight into how hybrid sterility may arise. Specifically, the activity of Msh2 has the potential to jeopardize chromosome segregation, risking the formation of aneuploid gametes and rendering hybrids derived from distantly related individuals sub- or infertile—contributing to the sexual isolation of a population. MMR may therefore not only influence the rates of evolution, favouring more gradual changes to the gene pool by limiting the amount of genetic exchange that may occur between divergent homologues, but also serve to promote speciation over evolutionary time.

## Supplementary Material

gkaf1136_Supplemental_File

## Data Availability

Data and code used in this study are publicly available at https://doi.org/10.25377/sussex.29459084. Octad and tetrad sequences are publicly available at the NCBI Sequence Read Archive (accession numbers SRP151982, SRP152540, and SRP152953).
