# Supplementary material for: Mismatch repair disturbs meiotic crossover control in S. cerevisiae
Source: Nucleic Acids Res. 2025 Nov 13;53(21):gkaf1136. doi: 10.1093/nar/gkaf1136 (PMC12614220; doi:10.1093/nar/gkaf1136)
Supplement: gkaf1136_Supplemental_File [file gkaf1136_supplemental_file.pdf]

Figure S1

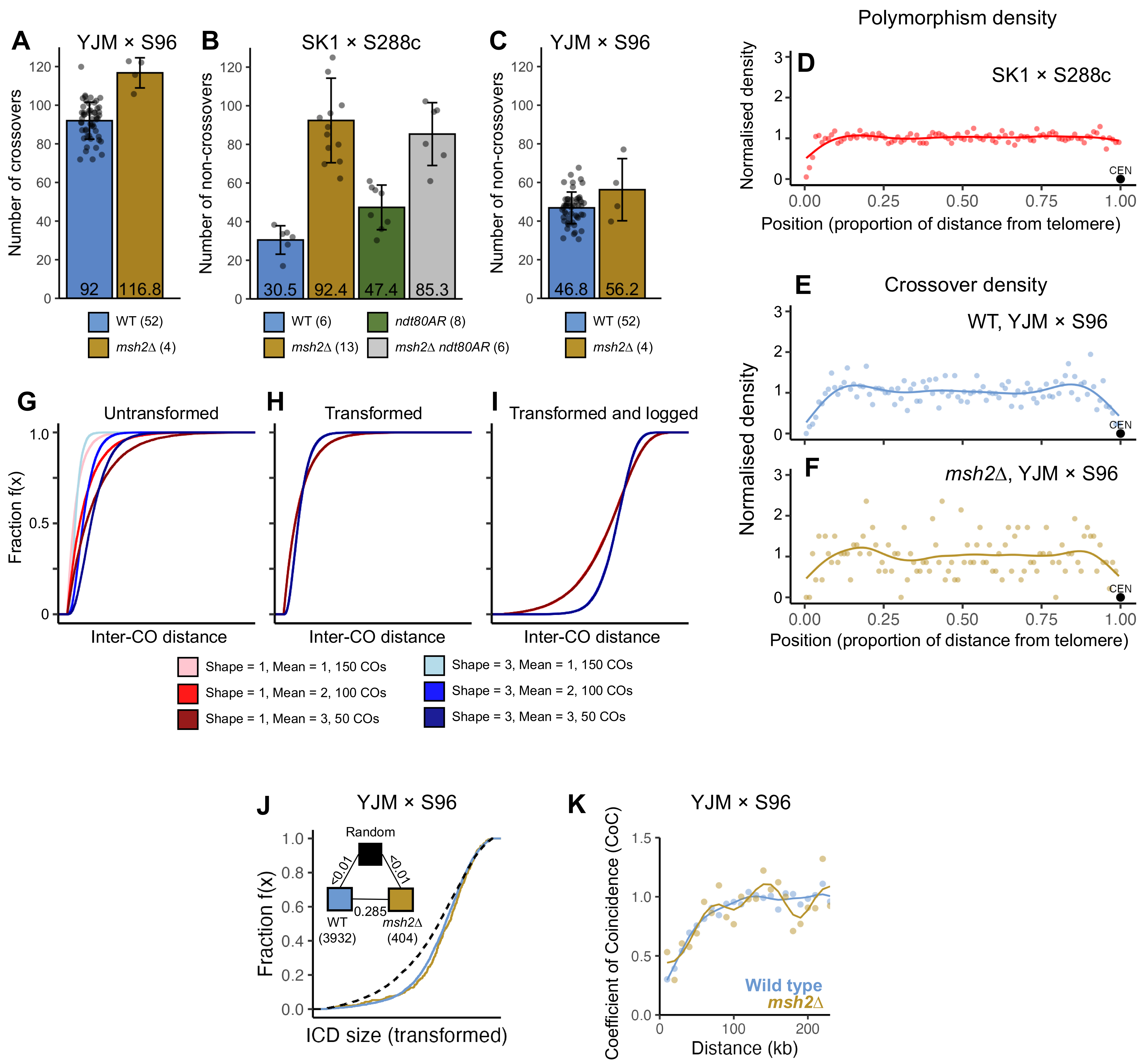

Figure S1. Non-crossover numbers and transformation of ICDs.
(A-C) Individual (grey circles) and average (bars with inset values) number of crossovers (A) and non-crossovers (B, C) per meioses for each genotype in SK1 × S288c (B) and YJM × S96 (A, C) hybrids. The number of individual meioses sequenced per genotype is indicated in brackets. Error bars represent standard deviation. (**D-F**) Polymorphism (D) and Crossover density (E, F) across chromosome arms. Chromosomes were split either side of the centromere and positions expressed as proportion of distance from telomere, normalizing for the size of chromosome arms. Densities were normalized by the highest observed density in each genotype. (**G-I**) Empirical cumulative distribution functions (eCDFs) of sampled gamma distributions showing the fraction of values at or below a given size, in untransformed values (G), values transformed by dividing by the mean of each distribution to correct for effects caused by variable event frequency (H) and log scaled transformed values to aid visualisation of the differing distributional spread caused by the alpha shape parameter (I). (**J**) Empirical cumulative distribution functions (eCDFs) showing the fraction of ICDs at or below a given size in the YJM × S96 background. ICDs were transformed (as in D-F; **Methods**) to correct for skews generated by differing CO frequencies. Black dashed lines represent randomised datasets generated via simulation to represent a state of no interference (**Methods**). Pairwise goodness-of-fit tests were performed between genotypes as indicated (triangular legend). P values: Two-sample KS-test. Numbers in brackets indicate the total number of ICDs in each genotype. (**K**) Coefficient of Coincidence curves across the stated genotypes (see **Figure 2A**). Paired intervals of the same distance across different chromosomes were pooled and averaged to calculate CoC.

Figure S2

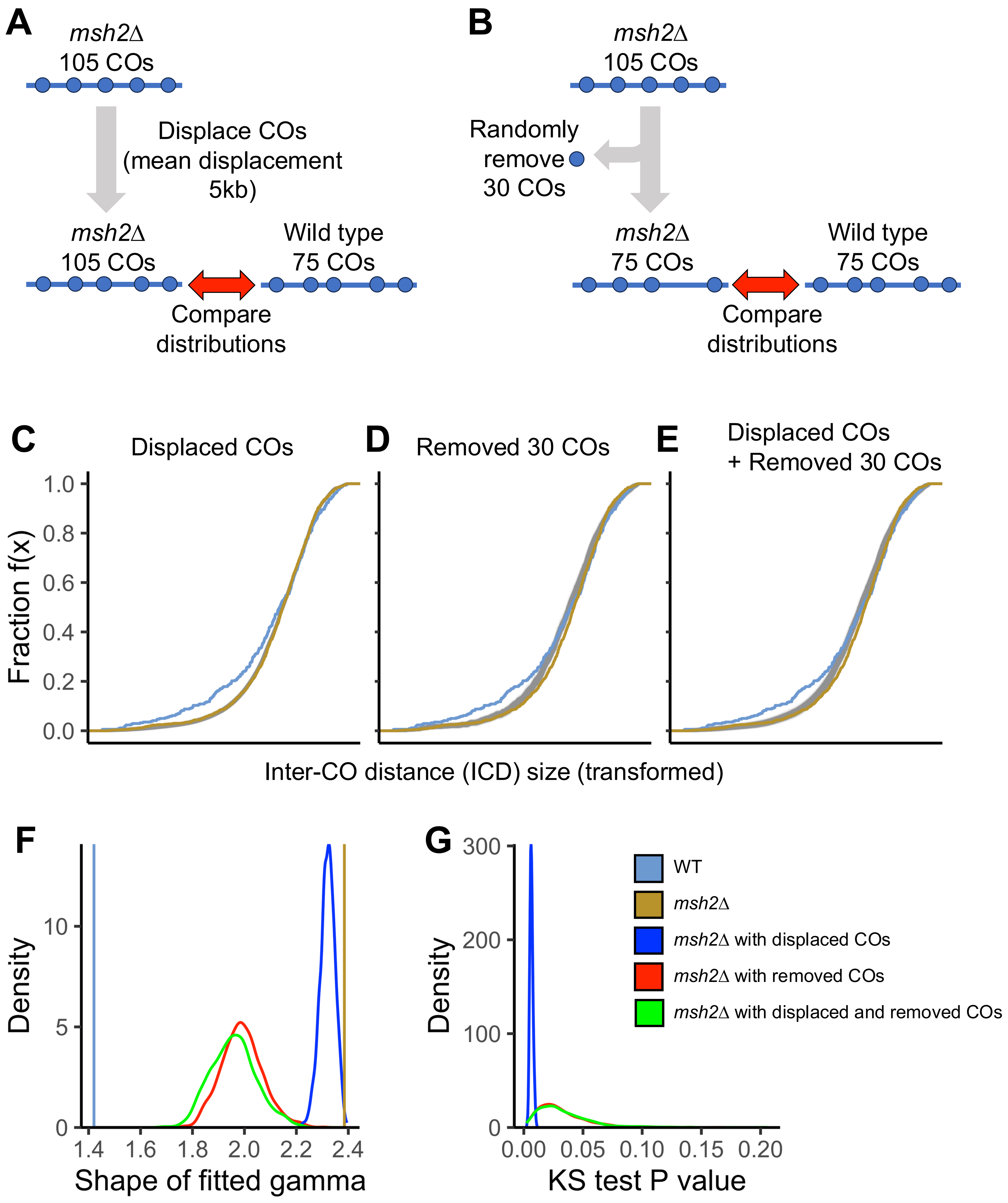

Figure S2. Differential mapping accuracies do not explain observed differences between wild type and *msh2*Δ strains. **(A)** *msh2*Δ crossovers were randomly displaced and ICDs recalculated. Displacement distances were modelled as a normal distribution, with mean 5kb and standard deviation 2.5kb. This process was repeated 1000 times. (**B)** 30 crossovers per meiosis from *msh2*Δ data were removed at random before recalculating ICDs and comparing to wild type. This process was repeated 1000 times. (**C-E)** Empirical cumulative distribution functions (eCDFs) showing the fraction of ICDs at or below a given size. Blue lines represent WT strains, gold *msh2*Δ. The grey lines represent *msh2*Δ data that has been altered, either by displacing COs randomly (C) (mean displacement 5 kb; see (A)), removing 30 random COs (D; see (B)), or both (E). ICDs were transformed (**Methods**) to correct for skews generated by differing CO frequencies. (**F, G)** Density curves of statistics performed on altered *msh2*Δ CO distributions, shape of best single gamma models fitted to altered ICDs (F) and Two-sample KS test *P* values between wild type and altered *msh2*Δ ICDs (G).

Figure S3

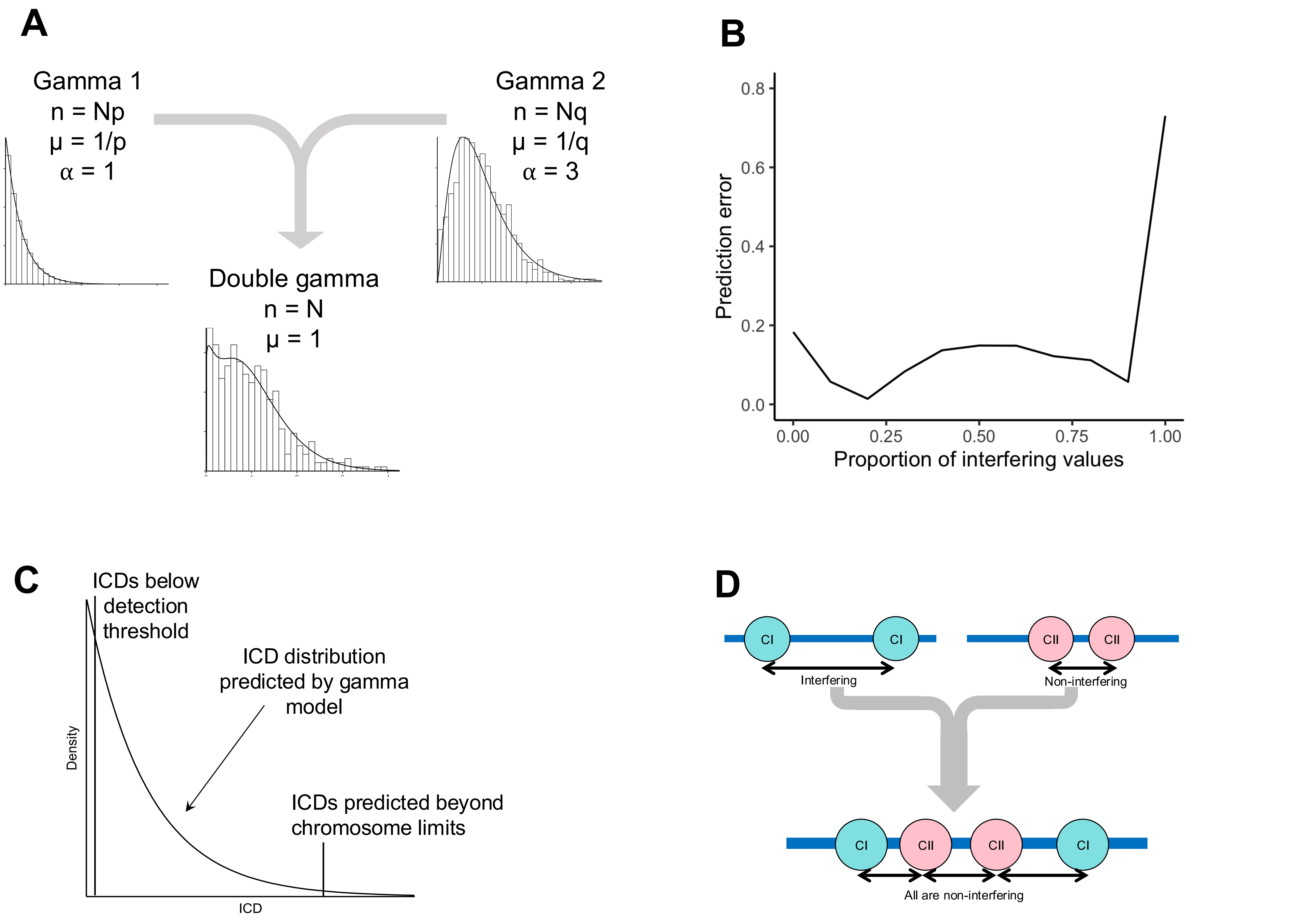

Figure S3. Mixed gamma models cannot deconvolve interfering and non-interfering ICDs in observed data. **(A)** Samples were taken from two different gamma distributions and mixed to mimic mixing of ICDs. (**B)** Error in mixed model fraction estimation against true proportion of interfering ICDs. (**C, D)** Constraints on ICDs observed compared to predictions from mixed gamma models. Range of impossible values predicted by unconstrained theoretical gamma distributions (C). Mixing interfering and non-interfering crossovers within the same genomic space can generate many additional non-interfering ICDs (D).

Figure S4

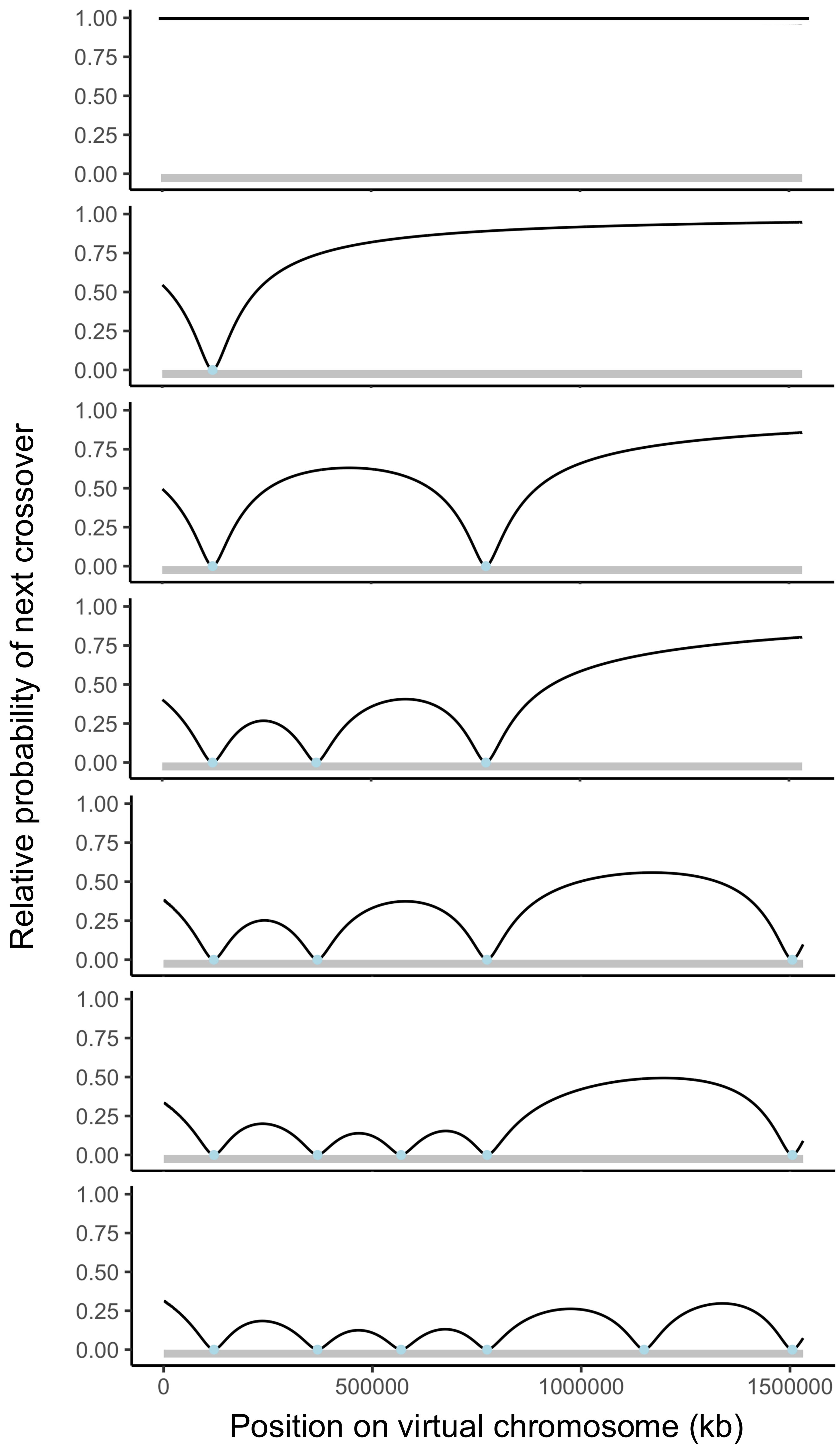

Figure S4. Simulation of crossover interference.
Example virtual chromosome plots from simulator showing sequential generation of interference pattern. Each wave of interference reduces the relative probability of future COs from forming in affected bins. The virtual chromosome here is 1.5 Mbp long, equivalent to *S. cerevisiae* chromosome IV.

Figure S5

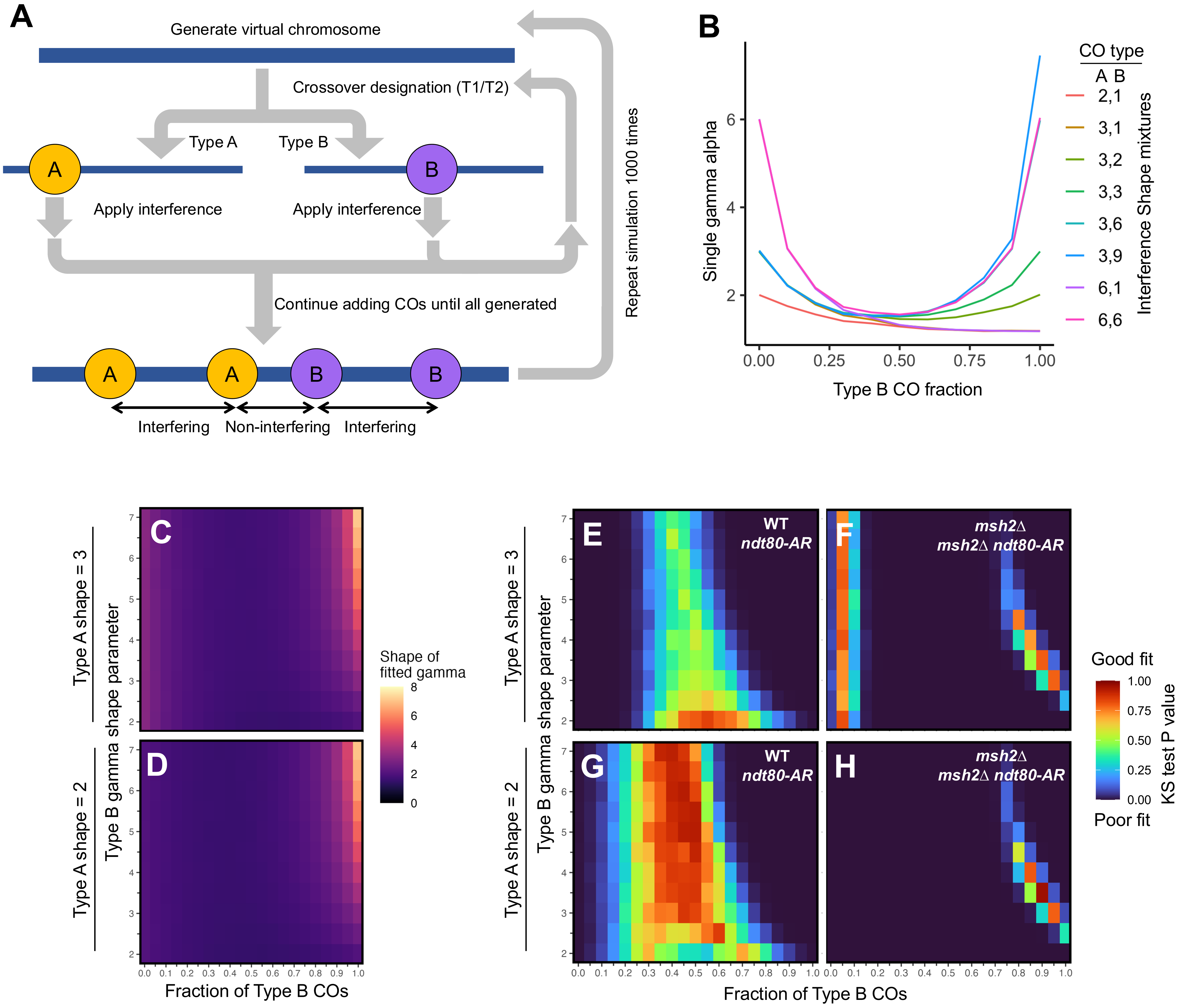

Figure S5. Minority crossover interference.

(A) Diagram of the potential types of ICDs that would be observed between type A (canonical) and B (minority) COs. **(B)** Variation in shape parameter of fitted gamma models to simulations of type A and B COs. **(C, D)** Coloured heatmap of the shape (α) of gamma distributions fitted to simulated ICD distributions. Each pixel represents a particular combination of parameter values: the shape of the type B interference function (Y axis) and type B CO % (X axis). Type A interference function was fixed at 3 (C) and 2 (D). **(E-H)** Coloured heat maps of *P* values (Two-sample KS test) between observed and simulated CO distributions expressed as eCDF curves for the indicated strains. *P* values >0.9 indicate good statistical fits. Each pixel represents a particular combination of parameter values: shape of type B interference function (Y axis) and type B CO fraction (X axis). Shapes of type A interference functions were set at 3 (E-F) and 2 (G-H) respectively. See main text and **Methods** for more details.

Figure S6

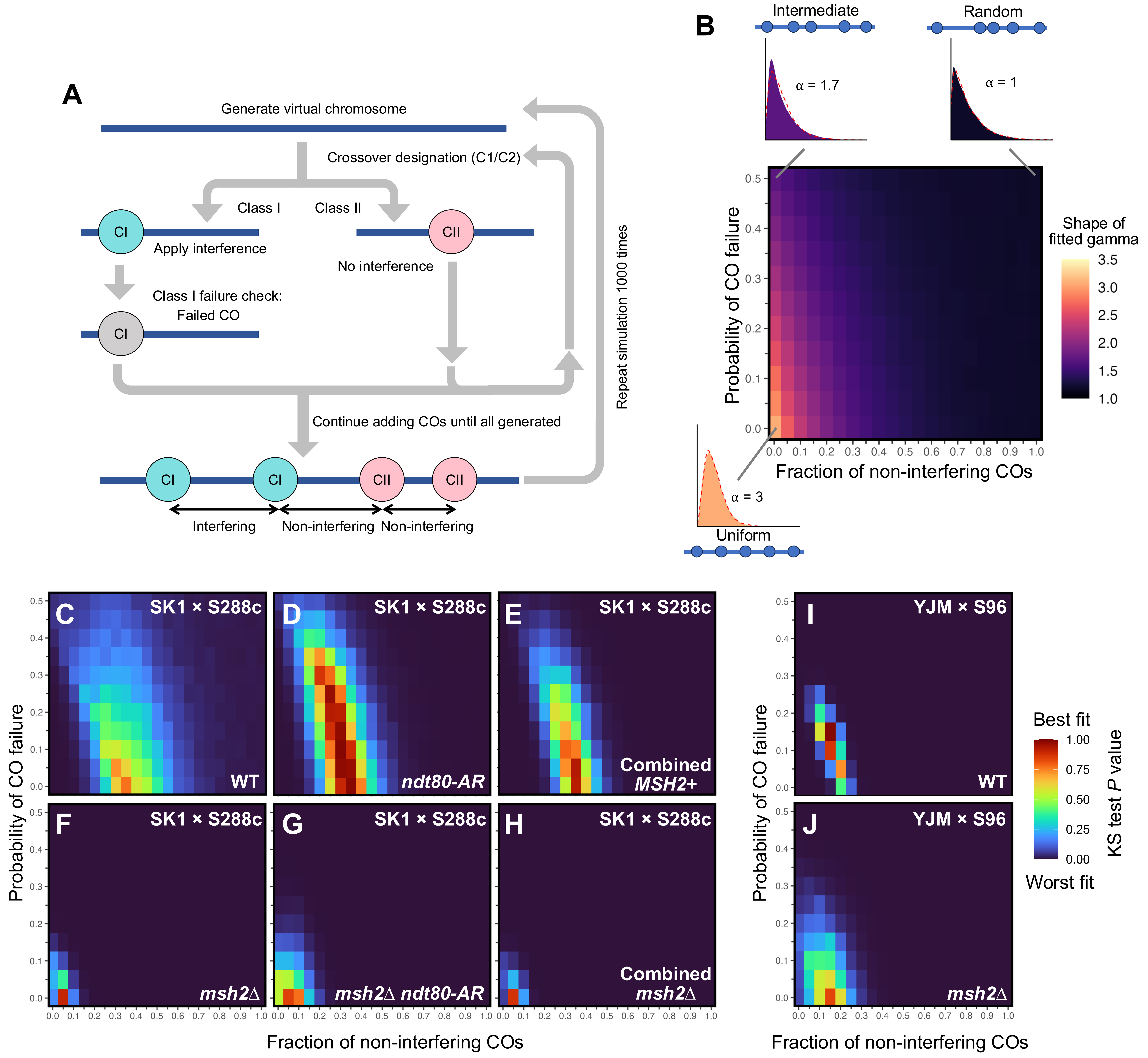

Figure S6. Crossover failure downstream of interference does not explain differences between wild-type and *msh2*Δ ICD distributions. **(A)** Schematic for simulation of random CO failure. (**B)** Coloured heatmap of the shape (α) of gamma distributions fitted to simulated ICD distributions. Each pixel represents a particular combination of parameter values: the probability of interfering CO failure (Y axis) and proportion of non-interfering COs (X axis). Smaller plots are probability density functions of example ICD distributions with representative fits, with parameters matching indicated pixels Red dotted lines represent gamma models fitted to simulated data. (**C-J)** Coloured heat maps of *P* values (Two-sample KS test) between observed and simulated CO distributions expressed as eCDF curves for the indicated strains and/or combined datasets. *P* values >0.9 indicate good statistical fits. Each pixel represents a particular combination of parameter values as in panel (B). See main text and **Methods** for more details.

Figure S7

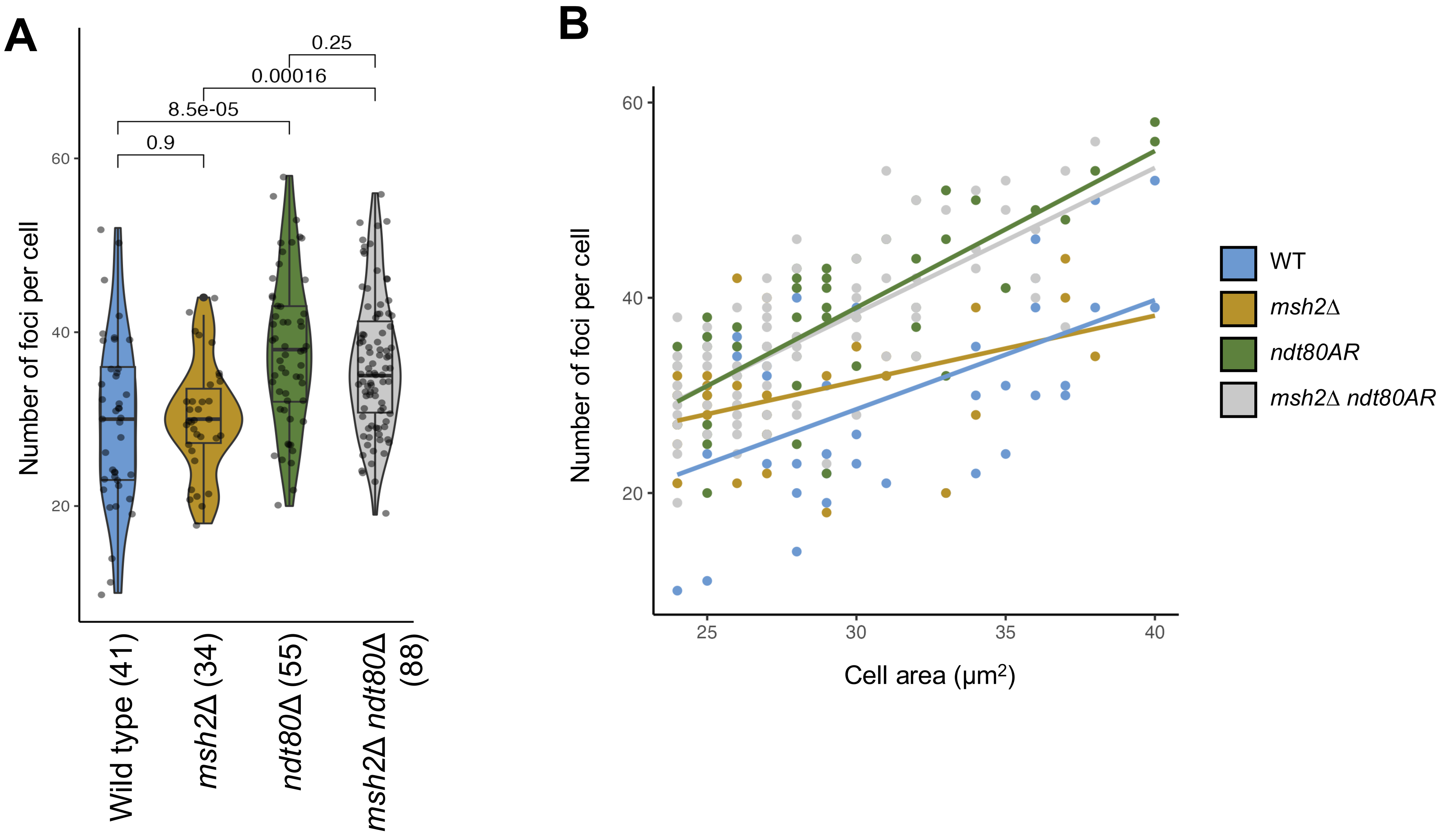

Figure S7. Detected Zip3 foci counts are positively correlated with DAPI-delimited nuclear-spread area. **(A)** Box-and-whisker plot showing Zip3 foci counts per square micron obtained from chromosome spreads of S288c × SK1 pachytene cells. Midlines denote median values, box limits are first and third quartile, whiskers are highest/lowest values within 1.5-fold of interquartile range. *P* values: Two-sample T-test. The total number of nuclei counted is indicated in brackets. (**B)** Scatter plot of Zip3 foci counts per cell against spread area delimited by the DAPI-positive signal for the indicated strains.

Figure S8

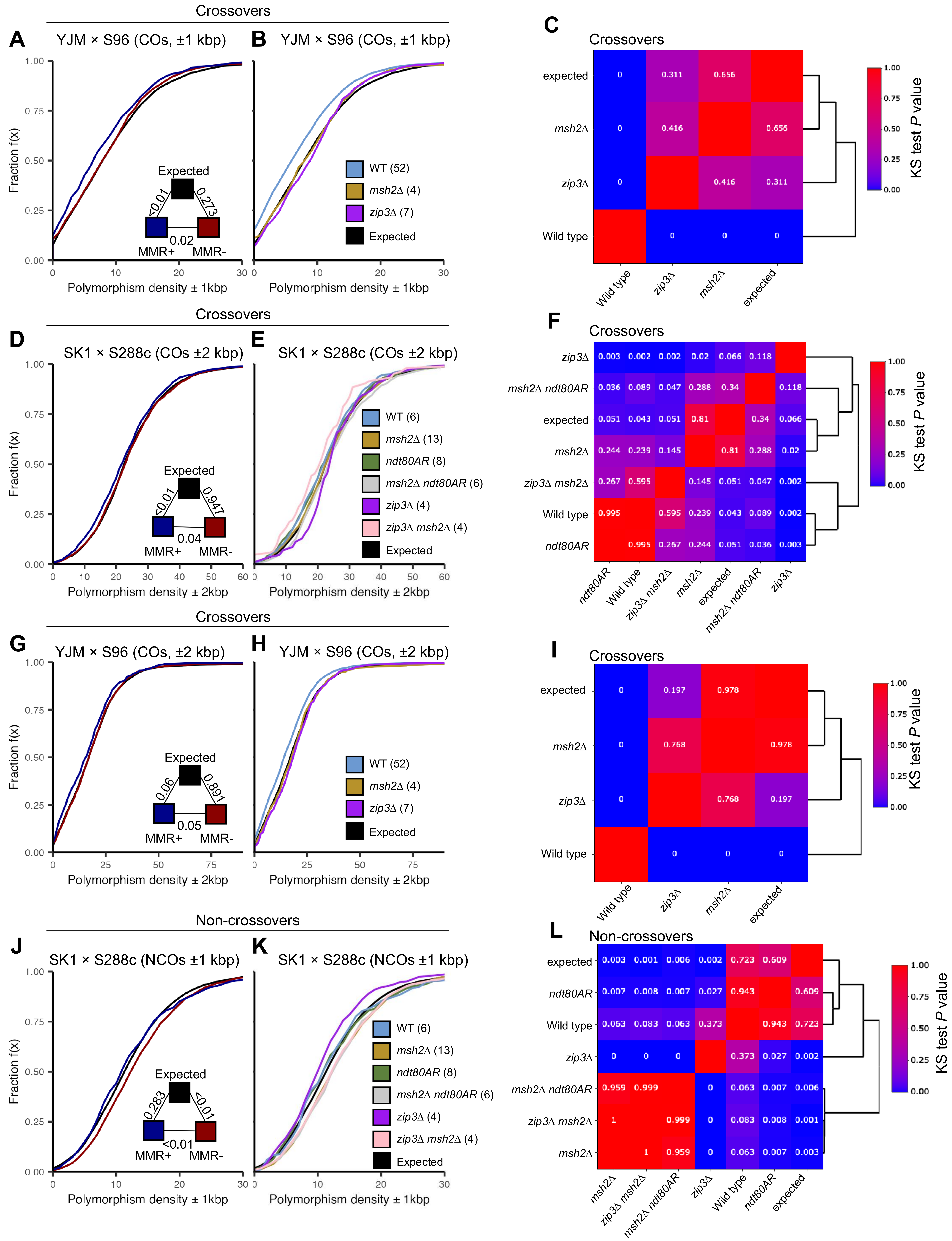

Figure S8. Polymorphism density in YJM × S96 hybrid, and in S288c × SK1 hybrid at wider windows and around non-crossovers. (**A**,**B**,**D**,**E**,**G**,**H**,**J**,**K**), Empirical cumulative distribution functions (eCDFs) showing the fraction of COs (A-I), or non-crossovers (J,K) that reside within a region of a given SNP/indel count in the indicated strains. In panel A,C,E,G, MMR+ indicates average of wild-type and *ndt80AR*; MMR- indicates average of *msh2Δ* and *msh2Δ ndt80AR*. (**C**,**F**,**I**,**L**) Similarity of polymorphism densities around COs (I-K), or NCOs (L) for the strains in the adjacent panels. Colours and numbers within each cell represent the KS test P value between indicated strains.

Figure S9

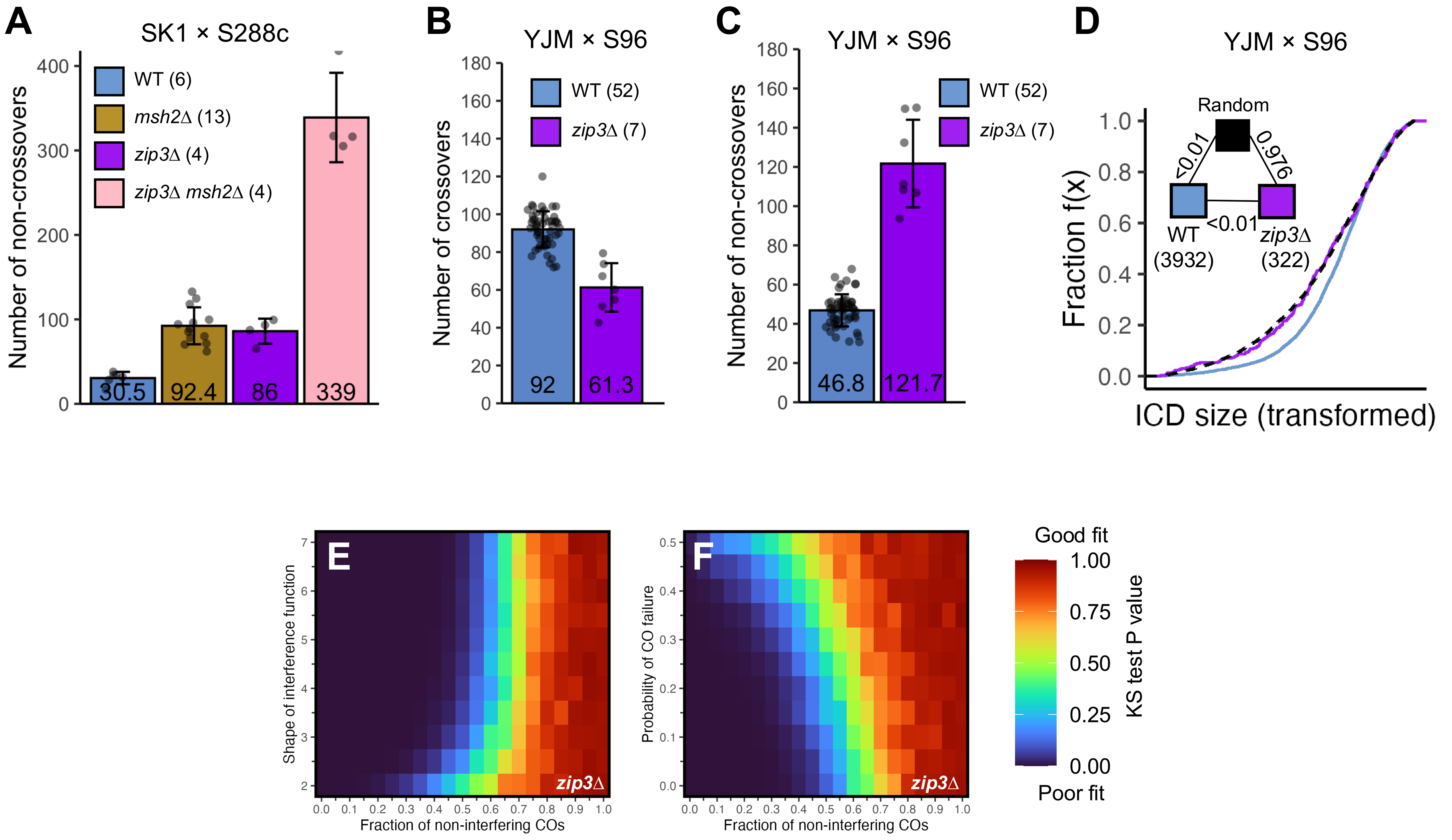

Figure S9. Effects of Zip3 on crossovers and noncrossovers.
(A-C) Individual (grey circles) and average (bars with inset values) number of NCOs (**A,C**) or COs (**B**) per meioses for each genotype. The number of individual meioses sequenced per genotype is indicated in tbrackets. Error bars represent standard deviation. (**D)** Empirical cumulative distribution functions (eCDFs) showing the fraction of ICDs at or below a given size. ICDs are transformed (**Methods**) to correct for skews generated by differing CO frequencies. Black dashed lines represent randomised datasets generated via simulation to represent a state of no interference (**Methods**). Pairwise goodness-of-fit tests were performed between genotypes as indicated (triangular legend). *P* values: Two-sample KS-test. Numbers in brackets indicate the total number of ICDs in each genotype. (**E, F)** Coloured heat maps of *P* values (Two-sample KS test) between observed and simulated CO distributions expressed as eCDF curves for the indicated strains. *P* values >0.9 indicate good statistical fits. Each pixel represents a particular combination of parameter values: shape of interference function (E; Y axis), maturation failure rate (F; Y axis) and non-interfering CO fraction (X axis). See main text and **Methods** for more details.

Figure S10

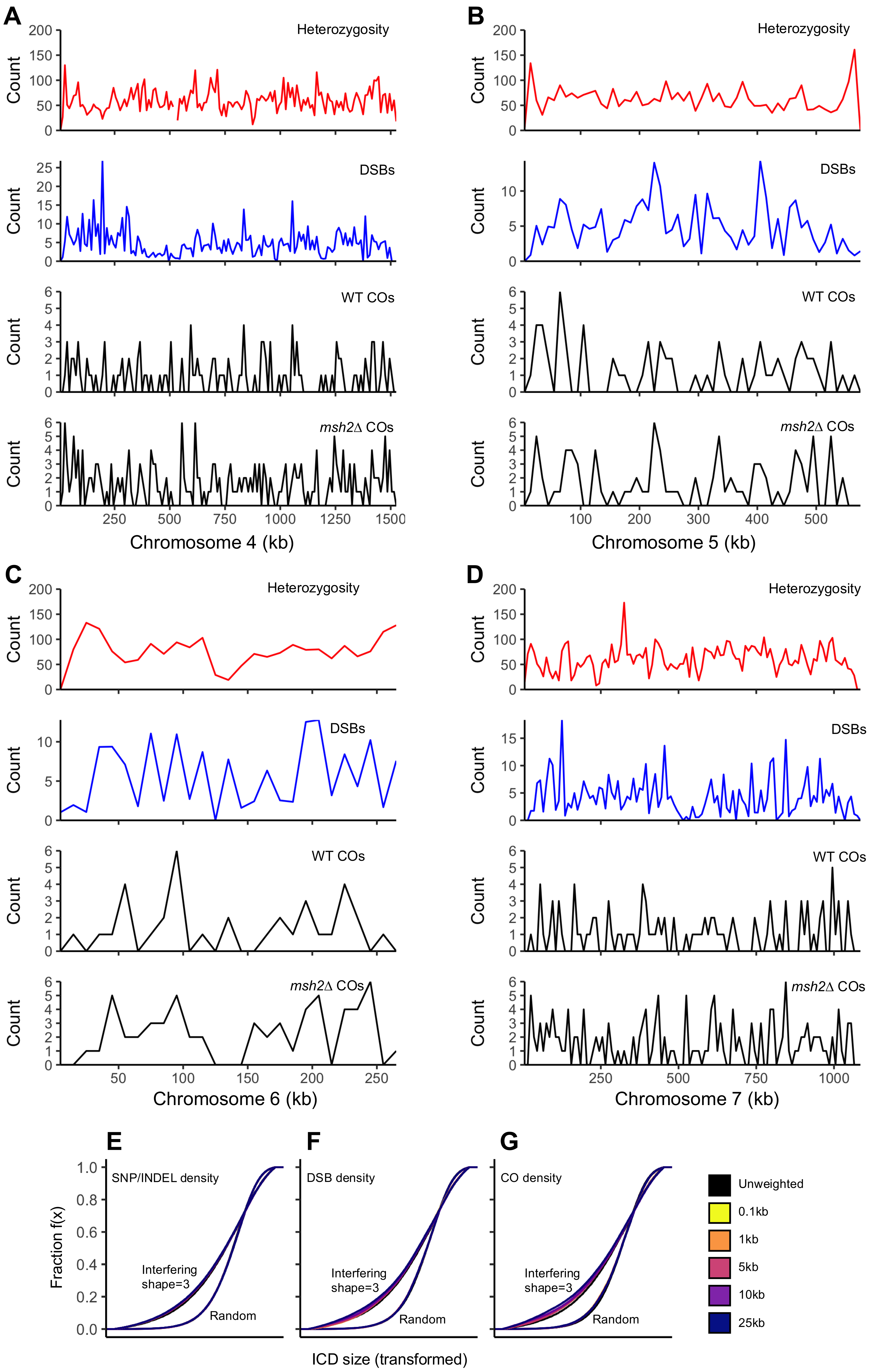

Figure S10. Impact on simulated crossover distributions of local deviations in density of heterozygosity, DSBs, and crossovers.
(A-D) Comparison of spatial distribution of population-average densities of heterozygosity, DSB formation [48], and CO formation in wild-type and *msh2Δ* cells for four representative chromosomes binned at 10 kb resolution. Although each chromosome has localised deviation from uniformity, each feature is spread relatively evenly across the length of each chromosome. DSB counts are the Spo11 read count per bp, with hotspots binned at 10 kb. (**E-G)** To test the impact that localised deviations in heterozygosity (E), DSB formation (F) and observed CO density (G) might have on relative distributions of COs, simulations of interfering (interference shape 3, 100% class I) and non-interfering COs (100% class II) were performed, but additionally by also weighting CO site selection by the relative amplitude of each parameter at varying levels of smoothing (0.1–25 kb). No significant changes in ICD distributions were observed, indicating that any non-uniformity in the distribution of these features does not significantly bias ICD distributions.

Figure S11

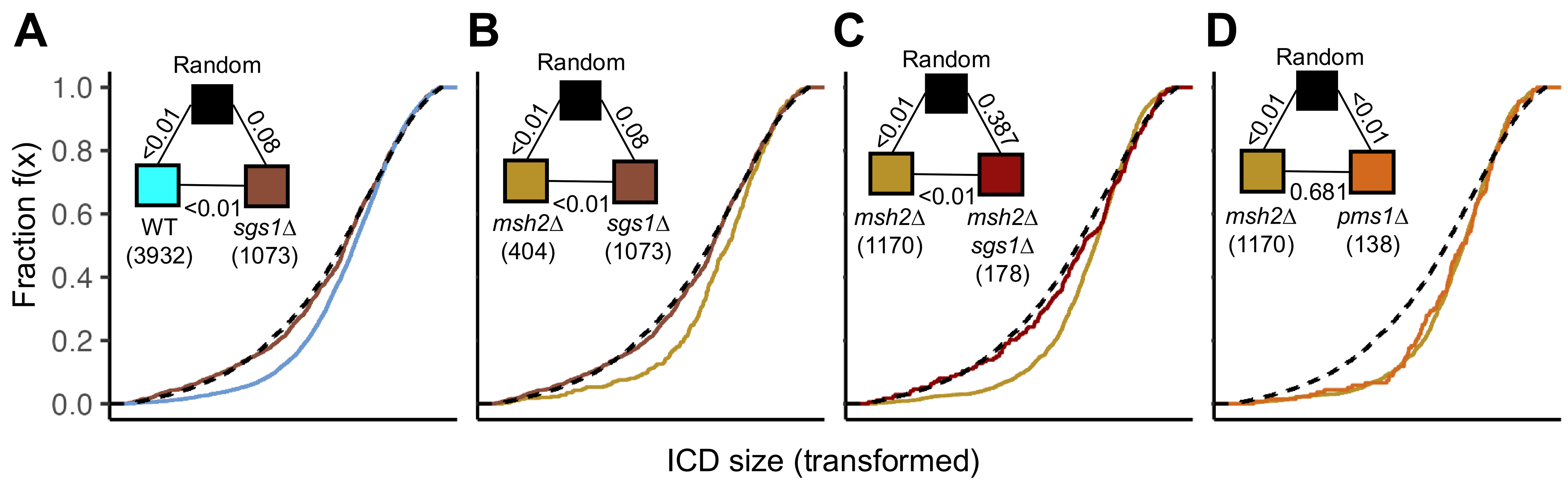

Figure S11. Effect of deletion of *SGS1* on the distribution of ICDs.
(A-D) Empirical cumulative distribution functions (eCDFs) showing the fraction of ICDs at or below a given size in the YJM × S96 hybrid (A, B) and the SK1 × S288c hybrid (C, D) for the indicated strains. ICDs were transformed (**Methods**) to correct for skews generated by differing CO frequencies. Black dashed lines represent randomised datasets generated via simulation to represent a state of no interference (**Methods**). Pairwise goodness-of-fit tests were performed between genotypes as indicated (triangular legend). *P* values: Two-sample KS-test. Numbers in brackets indicate the total number of ICDs in each genotype.

Table S1

Strain	Background	Mat	Genotype	Sequenced sample name
MJ513	SK1	a	*ho::LYS2 lys2Δ leu2 arg4Δ*	WT (TW)
MJ600	S288c	α	*ade8Δ*	WT (TW)
MJ43	SK1	α	*ho::LYS2 lys2Δ arg4Δ leu2Δ::hisG trp1Δ::hisG his4XΔ::LEU2 nuc1Δ::LEU2 PGAL1-NDT80::TRP1 ura3::pGPD1-GAL4(848)-ER::URA3*	*ndt80AR* (TN)
MC42	S288c	a	*ade8Δ ndt80Δ::KanMX*	*ndt80AR* (TN)
MC26	SK1	α	*ho::LYS2 lys2Δ ura3Δ arg4 leu2 msh2Δ::KanMX*	*msh2Δ* (OM)
MC49	S288c	a	*ade8Δ msh2Δ::KanMX*	*msh2Δ* (OM)
MC298	SK1	a	*ho::LYS2 lys2Δ ura3Δ arg4 leu2 trp1Δ::hisG ura3Δ::PGPD1-GAL4(848)-ER::URA3 PGAL1-NDT80::TRP1 msh2Δ::KanMX*	*msh2Δ ndt80AR* (OMN)
MC300	S288c	α	*ade8Δ ndt80Δ::KanMX msh2Δ::KanMX*	*msh2Δ ndt80AR* (OMN)
MC313	S288c	a	*ade8Δ zip3Δ::HphMX4*	*zip3Δ* (TZ)
MC322	SK1	α	*ho::LYS2 lys2Δ ura3Δ arg4 leu2 zip3Δ::HphMX4*	*zip3Δ* (TZ)
MC317	S288c	a	*ade8Δ msh2Δ::Kan zip3Δ::HphMX4*	*zip3Δ msh2Δ* (TMZ)
MC326	SK1	α	*ho::LYS2 lys2Δ ura3Δ arg4 leu2 msh2Δ::Kan zip3Δ::HphMX4*	*zip3Δ msh2Δ* (TMZ)

Supplementary Table 1. *S. cerevisiae* strains used in this study for tetrad/octad sequencing
All strains displayed are haploid, and were mated immediately prior to sporulation and tetrad dissection. Letters in brackets indicate the internal genotype labelling codes used to identify each dataset.

Table S2

Genotype	Cross	Background	Tetrads dissected	Mean spore viability	95% confidence interval	Viability pattern 4	3	2	1	0
WT	MJ600xMJ513	SK1xS288c	197	82.0	2.68	53.8	27.4	14.2	2.0	2.5
msh2Δ	MC49xMC26	SK1xS288c	149	73.0	3.56	40.3	25.5	21.5	11.4	1.3
ndt80AR	MJ43xMC42	SK1xS288c	86	70.4	4.83	36.1	25.6	25.6	9.3	3.5
msh2Δ ndt80AR	MC298xMC300	SK1xS288c	70	73.2	6.80	40.0	30.0	15.7	11.4	2.9
zip3Δ	MC322xMC313	SK1xS288c	106	46.2	4.75	13.21	18.9	31.1	13.2	23.6
zip3Δ msh2Δ	MC326xMC317	SK1xS288c	177	35.9	3.53	7.3	13.0	28.3	18.6	32.8

Supplementary Table 2. *S. cerevisiae* spore viability measurements. Viability is scored as the percentage of dissected spores that show visible growth after 48 hours incubation at 30ºC. To estimate how well the measurement represents the viability of the population, 95% confidence intervals are used. The colour scale used in column 5 indicates the mean spore viability reported for each sample: (Green > Yellow > Orange; indicating High > Medium > Low)
